# Development of PDMS Films Containing Thiamine Bromide and Sodium Iodide: Part 1—Matrix Characterisation and In Vitro Release

**DOI:** 10.3390/molecules31101588

**Published:** 2026-05-09

**Authors:** Zoya Farmazyan, Nelli Avagyan, Vigen Topuzyan, Emma Arakelova, Stepan Grigoryan, Mari Atabekyan, Susanna Grigoryan, Karen Khachatryan, Gohar Khachatryan

**Affiliations:** 1 The Scientific Technological Centre of Organic and Pharmaceutical Chemistry NAS RA, 26, Azatutyan Str., Yerevan 0014, Armenia; nelli.avagyan80@gmail.com (N.A.); vtop@web.am (V.T.); grigstepan@yahoo.com (S.G.); atmari@yandex.ru (M.A.); 2 Laboratory of X-Ray Structural Studies, National Polytechnic University of Armenia, 105, Teryan Str., Yerevan 0009, Armenia; emma_arakelova@yahoo.com (E.A.); susanna.grigorian@mail.ru (S.G.); 3 Laboratory of Nanotechnology and Nanomaterials, Faculty of Food Technology, University of Agriculture in Krakow, Al. Mickiewicza 21, 31-120 Krakow, Poland; karen.khachatryan@urk.edu.pl; 4 Department of Food Analysis and Quality Assessment, Faculty of Food Technology, University of Agriculture in Krakow, Al. Mickiewicza 21, 31-120 Krakow, Poland

**Keywords:** thiamine bromide, sodium iodide, PDMS films, Raman mapping, confocal Raman depth profiling, XRD, in vitro release, silicone matrix

## Abstract

This article represents the initial preformulation stage of a broader project aimed at evaluating polysiloxane films as carrier matrices for the TIODIN components thiamine bromide (ThBr) and sodium iodide (NaI). The specific gap addressed in this first part is the lack of information on how these two highly water-soluble crystalline salts are incorporated into hydrophobic crosslinked PDMS-based matrices, how they are distributed within such films, what solid-state forms and interactions may arise, and how these features relate to their release behaviour. Crosslinked films were prepared from α,ω-dihydroxypolydimethylsiloxane (PDMS–OH) of different viscosities, tetraethoxysilane (TEOS), and Sn(Oct)_2_ as a catalyst. Raman spectroscopy, confocal Raman depth profiling, and X-ray diffraction showed that the films contain both individual ThBr and NaI crystallites and mixed crystalline domains consistent with partial ThBr/NaI association and/or iodide-exchanged phases. The fraction of such mixed domains was higher in films prepared from lower-viscosity PDMS–OH than in films based on higher-viscosity PDMS–OH, and depth profiling extended this trend into the accessible near-surface layers from both film sides. Release into physiological saline, used here as a simple comparative aqueous release medium, remained low, reaching approximately 9% for ThBr and 20% for NaI after 72 h, while film swelling was minimal, approximately 1–1.5%. These findings are consistent with restricted water penetration and diffusion-limited release from hydrophobic, weakly swelling matrices. Because this first part of the study is restricted to matrix characterisation, depth profiling, and release into saline, the present results should be regarded as preformulation data. They do not demonstrate skin permeation, therapeutic transdermal performance, or suitability as a complete patch dosage form but establish baseline structural and release characteristics for a planned Part 2 focused on more application-oriented film optimisation, including properties required for future transdermal patch development.

## 1. Introduction

Transdermal drug delivery systems (TDDSs) remain attractive because they offer a non-invasive route of administration, can reduce dosing peaks, and may improve patient comfort and adherence relative to repeated injections. At the same time, successful transdermal delivery is restricted to a relatively small set of compounds with suitable physicochemical properties [1,2].

Silicone-based materials are widely used in patch technology, either as matrix-forming components or as pressure-sensitive adhesives. In the present work, however, the object of study is the drug-loaded silicone matrix itself. Complete patch construction, including an adhesive layer/backing and patch-performance parameters such as tack, skin adhesion, and skin compatibility, is outside the scope of this first part [3,4,5,6].

TIODIN is an aqueous injectable formulation containing thiamine bromide (ThBr) and sodium iodide (NaI). Developing a non-invasive alternative to repeated intramuscular administration is clinically attractive, and unlike oral administration, a transdermal approach could in principle avoid gastrointestinal exposure and first-pass metabolism [1,2]. Nevertheless, both ThBr and NaI are highly hydrophilic, predominantly ionised compounds and therefore intrinsically challenging candidates for passive transdermal delivery across the stratum corneum [2,7].

For this reason, the current article is framed as Part 1 of a broader study and focuses on preformulation questions: how ThBr and NaI are incorporated into crosslinked PDMS-based matrices, whether they remain as separate crystallites or form mixed domains, how these structural features are distributed across the accessible near-surface regions of the films, and how they correlate with release into a simple aqueous medium.

Accordingly, the aim of Part 1 is to characterise the morphology and phase behaviour of PDMS–OH/TEOS films containing ThBr and NaI using Raman spectroscopy, confocal Raman depth profiling, and XRD and to identify matrix-related variables that may influence subsequent formulation optimisation. The present study therefore addresses matrix physicochemistry and comparative in vitro release, rather than skin permeation performance itself. The term “film” is used throughout this article to describe the investigated PDMS-based matrices, whereas classification as a functional transdermal patch dosage form is intentionally not made at this stage. A subsequent Part 2 is planned to address the more application-oriented aspects required before such films can be considered as components of a transdermal patch system, including formulation optimisation, handling/adhesion-related properties, and dedicated permeation testing.

## 2. Results and Discussion

### 2.1. Characterisation of NaI, ThBr, and ThBr/NaI-P by FTIR, Raman Spectroscopy, and XRD

#### 2.1.1. FTIR and Raman Spectra of ThBr and NaI

To the best of our knowledge, there are no published FTIR or Raman spectra of thiamine bromide (ThBr); only detailed data for thiamine chloride (ThCl) are available [8]. Notably, that work does not describe bands below 200 cm^−1^.

The FTIR and Raman spectra of ThBr and NaI recorded in this work are shown in the Supplementary Materials (Figures S1a–c and S2). The absorption bands in the FTIR and Raman spectra of ThBr closely correspond to those reported for ThCl [8]. Comparison of the spectra shows that the 2500–3600 cm^−1^ region, associated with stretching vibrations of amino and hydroxyl groups, is more informative in the FTIR spectra of ThBr, whereas the Raman spectra clearly display bands in the 360–50 cm^−1^ region that are not observed in the FTIR spectra.

Since ThBr and NaI are crystalline compounds, the low-frequency region of the Raman spectra is particularly important for this study. It corresponds to external (lattice) vibrational modes (typically from about 200 down to 5 cm^−1^) and is highly sensitive to the crystal structure and polymorphic form. The intensities of these low-frequency bands are comparable to, or even higher than, the most intense high-frequency bands. It is well known that even small changes in the molecular configuration within the crystallographic unit cell can significantly affect the energies of these lattice modes [9].

#### 2.1.2. FTIR and Raman Spectra of ThBr/NaI-P

When preparing ThBr/NaI-P powder from aqueous solutions of ThBr and NaI, halide exchange in solution (ThBr + NaI ⇌ ThI + NaBr) may occur, with formation of thiamine iodide (ThI) [10,11]. Subsequent crystallisation can lead to Na(I,Br) mixed salts, and/or their hydrates (NaI/NaBr·xH_2_O), as well as new thiamine salts with iodide anions (ThI, including possible ThBr/ThI hydrates or polymorphs). In addition, hydrogen bonding between OH and NH_2_ groups and halide anions (O–H⋯I^−^, N–H⋯I^−^) may be formed.

**Figure 1:**
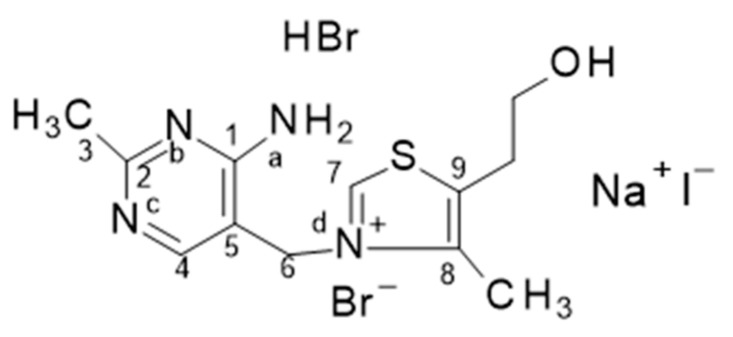
Formulation of ThBr/NaI-P powder.

Consequently, the FTIR and Raman spectra of the ThBr/NaI-P are expected to show changes in the vibrations of the main functional groups in the thiamine molecule. For example, substitution of Br by I in thiamine may lead to changes in the thiazole (R5) and pyrimidine (R6) rings; in the S–C_7_–Nd, C_7_NdC_8_, C_5_C_6_Nd, and C–O bonds; and in the NH_2_, OH, and CH_2_–CH_2_–OH groups (Figure 1).

Table S1 (Supplementary Materials) summarises the experimental FTIR and Raman wavenumbers of the main characteristic groups in ThBr and NaI and the corresponding changes observed in the powder ThBr/NaI-P. In the FTIR spectrum of ThBr/NaI-P, the OH stretching band at 3505 cm^−1^ in ThBr and the NH stretching band at 3440 cm^−1^ merge into a broad band with a maximum at about 3500 cm^−1^, whilst the OH deformation mode in the Raman spectrum shifts from ≈244 to ≈250–256 cm^−1^. These changes are consistent with the formation of hydrogen bonds and are accompanied by shifts in the C–O stretching region in both FTIR and Raman spectra. The NH_2_ stretching band at 3440 cm^−1^ in the FTIR spectrum is affected, whereas the NH_2_ bending modes (1656 cm^−1^ in FTIR and 1648 cm^−1^ in Raman) remain essentially unchanged. Only small shifts are observed in the out-of-plane deformation modes NH(NH_2_) at ≈540–580 cm^−1^.

In the thiazole ring (R5), both FTIR and Raman spectra show changes in the C–S stretching vibrations. The FTIR band at 867 cm^−1^ in ThBr appears at 882 and 897 cm^−1^ in the ThBr/NaI-P, and the Raman bands at 867.1 and 854.6 cm^−1^ in ThBr are transformed into a band at about 880 cm^−1^. More pronounced changes are observed in the out-of-plane deformation modes involving R5 and C–S: the band at 289.3 cm^−1^ assigned to C_5_–C_6_–Nd deformation and the band at 215.7 cm^−1^ assigned to the out-of-plane deformation C_7_–S–C_9_ disappear in the ThBr/NaI-P. Changes in the thiazole and pyrimidine ring breathing modes (R5 breath and R6 breath) are clearly evident in the FTIR spectra, whereas the corresponding Raman modes are only slightly affected.

In the low-frequency Raman region (<200 cm^−1^), NaI exhibits bands at 112.3, 76.8, and 55.5 cm^−1^, whereas ThBr shows bands at 112.3, 89.9, and 73.4 cm^−1^. In contrast, the ThBr/NaI-P does not display a simple superposition of these bands but shows new bands at 201.6, 164.4, 110.1, 84.3, and 56.1 cm^−1^. The appearance of these new lattice phonon bands and their shifts relative to the individual components indicate modifications of the crystalline lattice of thiamine and the formation of mixed or transformed crystalline phases in the ThBr/NaI-P.

These changes in the crystal structure of the ThBr/NaI powder are further confirmed by X-ray diffraction data (see Section 2.1.3).

#### 2.1.3. XRD Patterns of ThBr, NaI, and ThBr/NaI-P

**Figure 2:**
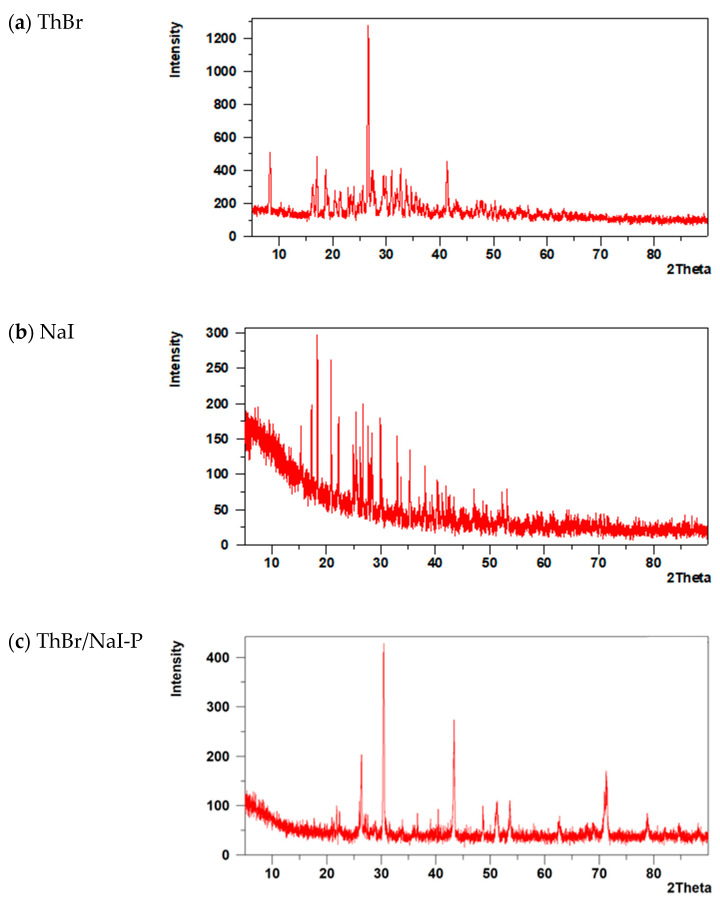
XRD patterns: (**a**) ThBr, (**b**) NaI, and (**c**) ThBr/NaI-P.

The X-ray diffraction (XRD) patterns of NaI, ThBr, and the recrystallised ThBr/NaI-P are shown in Figure 2a–c.

The characteristic diffraction peaks of ThBr, NaI, and their mixture (ThBr/NaI-P) are summarised in Tables S2, S2a, and S2b, respectively (Supplementary Materials).

In the XRD pattern of the ThBr/NaI-P, several reflections coincide with or are close to those of the individual components. For example, the peaks at 2θ = 26.37° (*d* = 3.377 Å) and 30.35° (d = 2.943 Å) are consistent with the main reflections of both NaI (26.25° and 29.93°) and ThBr (26.51° and 29.92°), indicating the presence of crystalline domains similar to the parent ThBr and NaI phases.

At the same time, a number of peaks in the ThBr/NaI-P are shifted relative to the corresponding NaI reflections. Peaks at 2θ = 51.20°, 53.58°, 62.63°, 69.04°, 70.91°, and 71.27° (*d* ≈ 1.78–1.32 Å) do not exactly match the positions reported for pure NaI but remain NaI-like. These shifts can be attributed to the formation of mixed Na(I,Br) salts and/or their hydrates, in which partial substitution of halide ions and incorporation of water molecules slightly distort the NaI lattice.

Most notably, the ThBr/NaI-P shows two additional reflections at 2θ = 78.87° (*d* = 1.213 Å) and 84.61° (*d* = 1.144 Å), which have no analogues in the XRD patterns of either pure ThBr or pure NaI. These new high-angle reflections are tentatively assigned to a new crystalline phase, most probably hydrated Na(I,Br) and/or mixed clusters containing Na(I,Br) and thiamine bromide/iodide species (Na(I,Br) + Th(I,Br)·(±H_2_O)). This interpretation is consistent with the changes in the Raman spectra of the ThBr/NaI-P discussed in Section 3.1.2, where the appearance of new low-frequency lattice modes also indicates modifications of the crystalline lattice and the formation of mixed or transformed phases.

Thus, the combined XRD and Raman spectroscopy data suggest that the ThBr/NaI-P obtained by crystallisation from aqueous solution contains not only a simple physical mixture of ThBr and NaI crystals but also a combination of residual ThBr and NaI phases, NaBr and hydrated Na(I,Br)-type salts, and possibly mixed thiamine bromide/iodide phases (ThBr/ThI).

These interactions between ThBr and NaI are observed in the powder obtained by crystallisation of an aqueous solution of thiamine salt with sodium iodide at a molar ratio of 1:2.

An important question for this work is whether similar interactions between thiamine bromide (ThBr) and sodium iodide (NaI) can occur during the formation of the polysiloxane matrix films containing ThBr and NaI, where only trace amounts of water are present. Tetraethoxysilane (TEOS) is known to undergo hydrolysis in the presence of atmospheric moisture, and water may also be generated in the films as a product of TEOS hydrolysis–self-condensation and/or its condensation with PDMS–OH. Therefore, even small amounts of water in the polysiloxane films may be sufficient to trigger partial halide exchange and the formation of mixed Na(I,Br) and thiamine bromide/iodide phases analogous to those detected in the ThBr/NaI powder. This hypothesis is further examined in the XRD and Raman studies of the composite polysiloxane films presented below.

### 2.2. Films PDMS-p, 26M, 36M

#### 2.2.1. Raman Spectra of PDMS-p Film

A crosslinked PDMS-p film (pure matrix without ThBr and NaI) was synthesised as a reference sample for comparison with the composite films in which both salts are present. The key reactions involved in the synthesis of crosslinked polydimethylsiloxane (PDMS) via condensation between PDMS–OH and tetraethoxysilane (TEOS), catalysed by stannous octoate (Sn(Oct)_2_), have been investigated in detail elsewhere [12,13,14,15] and are summarised in the Supplementary Materials.

As a result of these reactions, an in situ SiO_2_ phase may form within the PDMS matrix, which may appear as nanoclusters or as a continuous inorganic network depending on the TEOS content and water availability.

An important feature of PDMS film formation is that TEOS hydrolysis can proceed even in the absence of deliberately added water because of atmospheric moisture. Therefore, the formation of a SiO_2_ phase may initially occur preferentially at the surface in contact with air, as reported elsewhere [12,16,17,18,19]. This can give rise to structural inhomogeneity across the film thickness and to differences in morphology between the two surfaces—the air side and the substrate side.

Raman spectra of the PDMS-p film

Raman spectra of the PDMS-p film (air side, AS; substrate side, SS) are shown in Figure S4 (Supplementary Materials). The positions of the main bands (cm^−1^) for both surfaces are identical (Table S3, Supplementary Materials) and correspond to the known vibrational modes of PDMS reported in the literature [13,14].

#### 2.2.2. XRD Patterns of the PDMS-p Film

**Figure 3:**
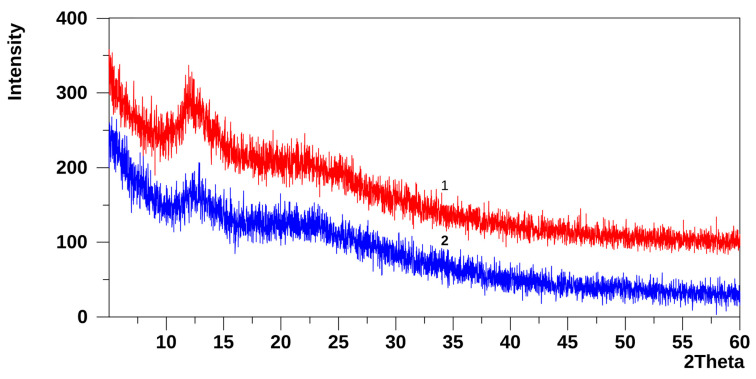
XRD patterns of the PDMS-p film: (1) air side; (2) substrate side.

XRD patterns of the PDMS-p film are shown in Figure 3 for the air side and the substrate side of the film. Both sides of the PDMS-p film exhibit two broad amorphous halos with slightly different positions of their maxima: on the air side at 2θ ≈ 11.8°, with a weaker maximum at 2θ ≈ 22.2°, and on the substrate side at 2θ ≈ 12.5°, with the second, less intense maximum at about 2θ ≈ 23.1°.

Amorphous halos with maxima in the 2θ ≈ 11–12.5° region are characteristic of many PDMS films [15,20,21] and are usually associated with partially ordered domains formed by PDMS chains or chain segments. The broad diffraction maximum in the 2θ ≈ 22–25° region is attributed to short-range order within Si–O–Si networks in the amorphous film. Previously, we observed a similar halo at 2θ ≈ 23.2°, characteristic of Si–O–Si structures in a TEOS hydrolysate (TEOS xerogel), whose diffraction pattern consists of a single amorphous maximum at 2θ ≈ 23.2° [22].

**Figure 4:**
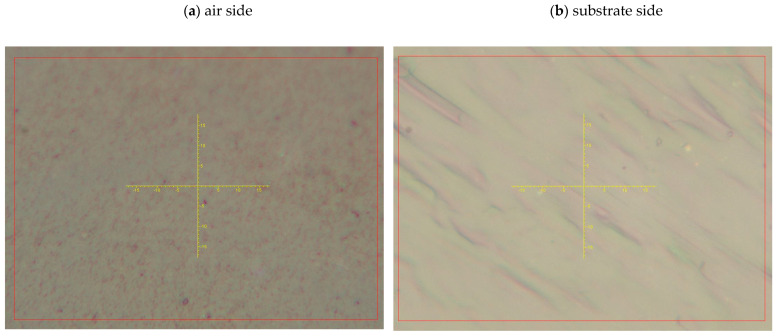
Optical images of the PDMS-p film surfaces: (**a**) air side; (**b**) substrate side.

Although the average Raman spectra and XRD patterns of both surfaces of the PDMS-p film are essentially the same, optical images obtained with the Raman microscope (Figure 4) reveal noticeable differences in morphology between the two surfaces. On the air side, elongated, lamellar, stripe-like domains oriented approximately parallel to one another are observed, presumably associated with the orientation and partial ordering of PDMS chain segments. In contrast, the substrate side shows smaller domains with only partial ordering.

The examination of the ThBr/NaI-P powder and of the PDMS-p film provides a basis for analysing possible interactions between ThBr and NaI during the formation of crosslinked PDMS films containing these salts. In addition to atmospheric moisture, which drives TEOS hydrolysis, water can be generated in situ as a product of TEOS hydrolysis–self-condensation and/or its condensation with PDMS–OH. In such a medium, not only network-forming reactions but also physicochemical interactions between the salts—similar to those observed in the ThBr/NaI-P powder, including partial ion exchange, association, and hydration—may occur.

### 2.3. Films 26M, 36M with ThBr and NaI

#### 2.3.1. Raman Spectra of Films 26M and 36M

Raman spectra of films 26M and 36M were recorded at several points on both film surfaces (air side and substrate side). These spectra reveal clear differences between the two surfaces for both films. On the substrate side of film 36M, only the characteristic PDMS bands are observed. In contrast, on the substrate side of film 26M, in addition to the PDMS bands, the main Raman bands of ThBr are detected at 1648, 945, 750, 1055, and 89 cm^−1^. On the air-side surfaces of both films, all characteristic bands of PDMS, ThBr, and NaI are present in the Raman spectra. Examples of spectra are shown in Figures S5 and S6 (Supplementary Materials).

On the air-side surfaces of both 26M and 36M, noticeable variations are observed between spectra recorded at different points, particularly in the low-frequency region below 200 cm^−1^. These changes are consistent with local differences in salt organisation and possible lattice modification relative to individual ThBr and NaI, analogous to the behaviour observed for the ThBr/NaI-P powder.

To understand these spectral differences at different points and to obtain information on the spatial distribution of salt domains in films 26M and 36M, Raman mapping was performed (Section 2.3.3).

#### 2.3.2. XRD Patterns of Films 26M and 36M

**Figure 5:**
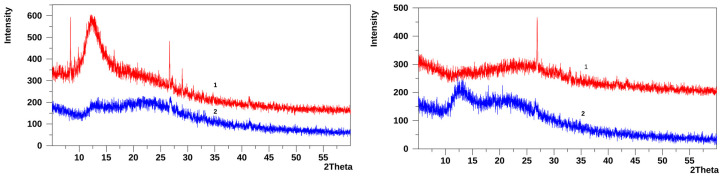
XRD patterns of films 36M and 26M: (1) substrate side; (2) air side.

The XRD patterns of films 26M and 36M (substrate side and air side) are shown in Figure 5. The patterns of both films are predominantly amorphous, similar to the reference PDMS-p film, but several distinct crystalline reflections are superimposed on the amorphous halos.

The positions and tentative assignments of the crystalline reflections for both sides of each film are summarised in Tables S4 and S5 (Supplementary Materials), in comparison with the reflections of individual ThBr and NaI.

For the air side of film 26M, several reflections in the 2θ ≈ 26.9–31.2° region can be assigned to overlapping contributions from ThBr- and NaI-like phases, based on comparison with the diffraction patterns of the individual salts (Tables S2 and S4). Additional reflections at 2θ = 32.95° and 41.62° correspond to NaI and ThBr, respectively. New reflections at 2θ = 36.41° and 43.45° do not have clear analogues in the XRD patterns of pure ThBr or NaI and likely originate from iodine-containing phases, such as mixed Na(I,Br) salts and/or thiamine iodide/bromide species (Th(I,Br)) and their hydrates.

On the substrate side of film 26M, the reflection at 2θ = 26.58° can be attributed to overlapping NaI/ThBr contributions, whereas the reflection at 2θ = 38.45° is consistent with Na(I,Br)-type phases. The low-angle reflection at 2θ = 11.73°, corresponding to a large *d*-spacing, may be associated with hydrated thiamine bromide/iodide species (Th(I,Br)·±H_2_O).

The air-side surface of film 36M is dominated by ThBr-like reflections at 2θ = 17.10°, 27.16°, 31.06°, and 41.38°, together with an overlapping NaI/ThBr region at about 2θ ≈ 26.65°. Two reflections at 2θ = 28.99° and 34.72° have no clear analogues in the patterns of pure ThBr or NaI and may correspond to new iodine-containing phases, such as ThI polymorphs/hydrates and/or Na(I,Br) salts. The reflection at 2θ = 47.72° can be assigned to NaI. On the substrate side of film 36M, reflections at 2θ = 26.75° and 41.45° can be assigned to ThBr-like phases (26.63° and 41.31° for ThBr).

In both films, the new reflections may also, at least in part, originate from associates between NaI and low-molecular-weight TEOS hydrolysis products (Na–O–Si–O-type environments involving surface Si–OH groups).

Taken together, the Raman and XRD data indicate that in both films 26M and 36M, interactions between ThBr and NaI similar to those observed in the ThBr/NaI-P powder partially occur during film formation. At the same time, the pattern of reflections suggests that in film 26M, there is a higher fraction of mixed ThBr/NaI clusters, whereas in film 36M, the salts are predominantly present as more isolated crystalline domains. It is likely that the lower viscosity of PDMS–OH in 26M (η ≈ 1000 cP) facilitates redistribution and partial co-crystallisation of ThBr and NaI during TEOS hydrolysis and crosslinking, whilst the higher viscosity of PDMS–OH in 36M (η ≈ 2000 cP) restricts this redistribution. Raman mapping provides additional information on the spatial distribution of these salt domains in films 26M and 36M (Section 2.3.3).

In the revised interpretation, these assignments are treated as tentative. The Raman and XRD data support the presence of NaI-like, ThBr-like, and mixed/iodide-modified salt domains, but they do not by themselves provide direct compositional proof of specific Na(I,Br), Th(I,Br), or hydrated phases. Throughout the discussion, these phase labels should therefore be read as working assignments rather than definitive structural identifications.

#### 2.3.3. Raman Spectra and Mapping of Films 26M and 36M (Air Side)

Raman mapping was carried out to assess whether ThBr and NaI are present in the films mainly as separate crystallites or as associated mixed phases. For each film, 3–4 regions on the air side were investigated. For each region, intensity maps were recorded for selected Raman bands: in the low-frequency region (<200 cm^−1^), for the internal vibrational modes of the pyrimidine and thiazole rings (750 cm^−1^, R6 breathing; 945 cm^−1^, R5 breathing), as well as for functional groups at 1648 cm^−1^ (NH_2_ deformation) and for C–O stretching bands in the 1000–1100 cm^−1^ region (Table S1, Supplementary Materials).

In this section, one representative region is discussed for each film; the remaining maps are presented in the Supplementary Materials.

In the spectrum of region 26M map 2, the low-frequency part (<200 cm^−1^) contains a weak band near 58–59 cm^−1^ and more intense bands at approximately 84 and 110 cm^−1^. These values are close to the set of low-frequency bands observed for the ThBr/NaI-P powder (84.3, 110.1, and 56.1 cm^−1^), whereas the individual salts show ThBr bands at 112.3, 89.9, and 73.4 cm^−1^ and NaI bands at 112.3, 76.8, and 55.5 cm^−1^. The C–O stretching bands at 1067 and 1042 cm^−1^ in this region also correspond to those of the ThBr/NaI-P powder.

**Figure 6:**
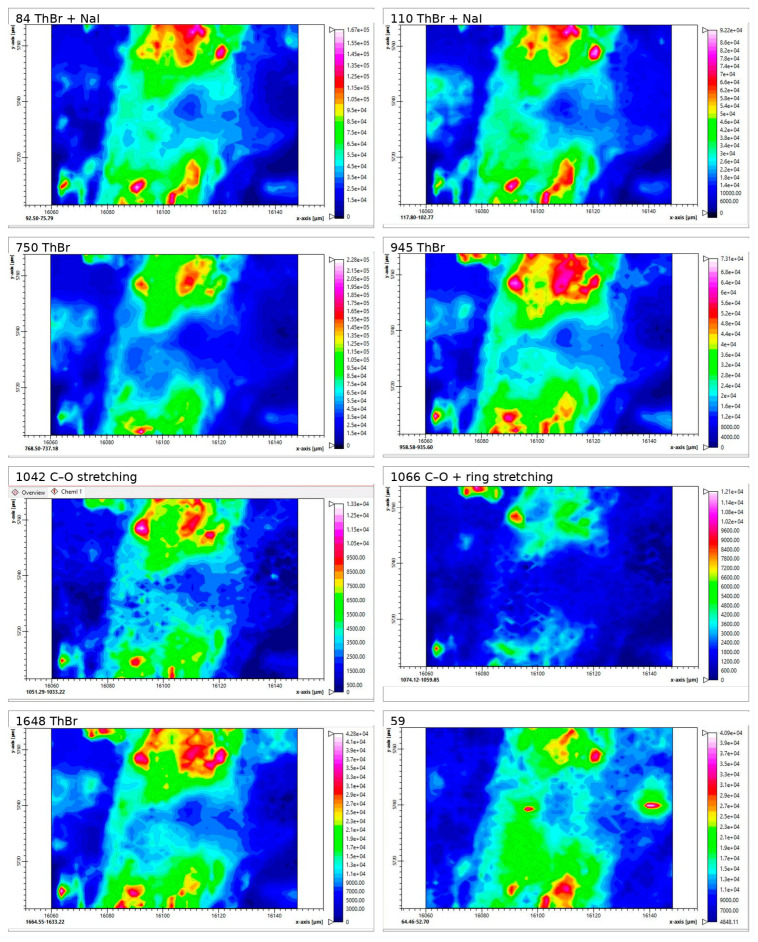
Raman mapping of film 26M (air side), region map 2 (selected bands: 59, 84, 110, 750, 945, 1042, 1066, and 1648 cm^−1^).

The intensity maps for the bands at 84 and 110 cm^−1^ (Figure 6) exhibit very similar spatial patterns and almost perfectly overlap with the maps of the internal thiamine modes at 750, 945, 1042, 1067, and 1648 cm^−1^. This colocalisation indicates that the low-frequency modes at 84 and 110 cm^−1^ are associated with thiamine-containing crystalline domains in which ThBr and NaI form mixed ThBr/NaI-type clusters.

At the same time, in the Raman maps at 1042 and 1067 cm^−1^ (C–O stretching), slight variations in relative band intensity and ratio (I_1042_/I_1067_) are observed across different domains. This suggests heterogeneity in the local environment of thiamine, for example in the orientation, degree of ordering, and hydrogen-bonding patterns in different domains.

The map for the weak band near 59 cm^−1^ in region 26M map 2 shows a pattern similar to those of the 84 and 110 cm^−1^ bands, suggesting that in this region, the 59 cm^−1^ band also has a contribution from ThBr/NaI-type associates, in addition to possible Si–O–Si-related modes.

Mapping of two additional regions on the air side of film 26M (maps 1 and 3) showed that domains with ThBr/NaI-P-type associates are also present in the second region, whereas the third region probably contains both mixed ThBr/NaI associates and individual ThBr crystallites (see the Supplementary Materials, Figure S6).

In the spectrum of region 36M map 2, the low-frequency range (<200 cm^−1^) shows bands at 73, 89, and 112 cm^−1^. These values correspond to the low-frequency bands of individual ThBr (73.4, 89.9, and 112.3 cm^−1^) and partly NaI (band at 112.3 cm^−1^) (Table S1, Supplementary Materials). Thus, in region 36M map 2, ThBr and NaI are predominantly present as individual salt crystallites rather than as mixed ThBr/NaI clusters characteristic of the ThBr/NaI-P powder.

**Figure 7:**
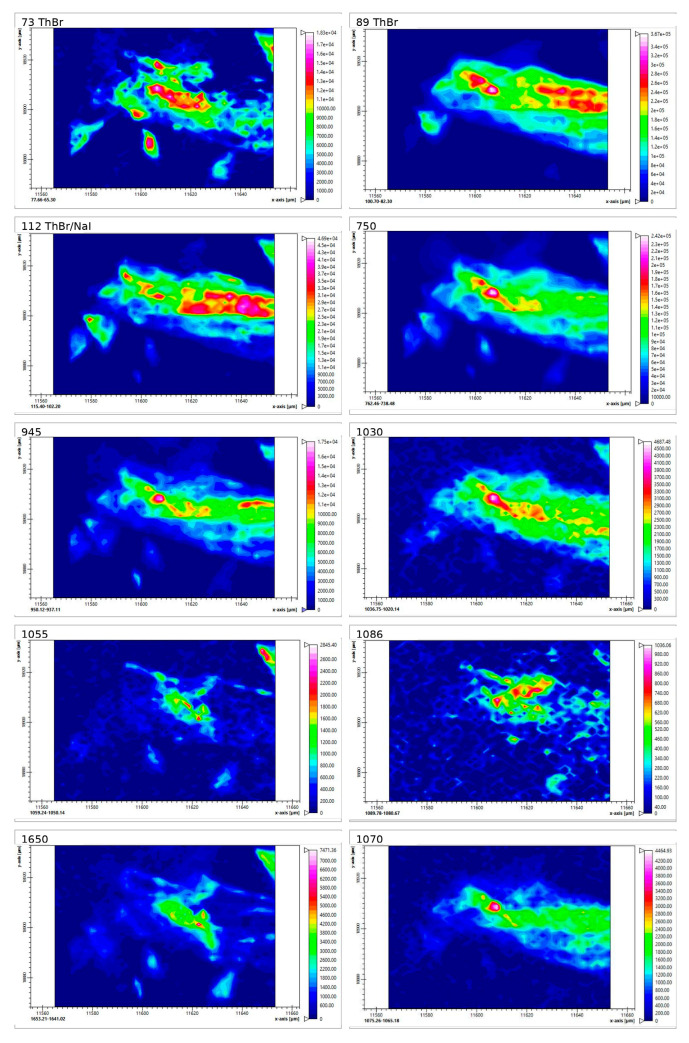
Raman mapping of film 36M (air side), region map 2 (selected bands: 73, 89, 112, 750, 945, 1030, 1055, 1070, 1086, and 1650 cm^−1^).

The Raman maps for the bands at 73, 89, and 112 cm^−1^ (Figure 7) exhibit patterns similar to those of the internal modes of thiamine at 750, 945, and 1650 cm^−1^ (R6 breath, R5 breath, and NH_2_ deformation), confirming that these low-frequency bands are associated with ThBr crystallites.

The maps for the C–O bands at 1030, 1055, 1070, and 1086 cm^−1^ show overlapping spatial distributions with the thiamine modes (750, 945, and 1650 cm^−1^) and with the low-frequency bands (73, 89, and 112 cm^−1^). This behaviour may reflect the involvement of hydroxyl and amino groups in intermolecular interactions (e.g., hydrogen bonding), as suggested by small shifts in the C–O and NH bands relative to individual ThBr (1086 vs. 1088 cm^−1^; 1650 vs. 1648 cm^−1^). Mapping of two additional regions (maps 1 and 3) showed similar patterns, indicating that over the studied areas, the salts in film 36M are predominantly present as individual ThBr and NaI crystallites (Supplementary Materials, Figure S7).

Taken together, these results indicate that in film 36M, the salt-containing domains are more isolated (predominantly, individual ThBr and NaI crystallites), whereas film 26M exhibits a more pronounced formation of mixed ThBr/NaI associates. These differences in the microstructure of the salt-containing domains are expected to influence the mechanism and kinetics of salt release from films 26M and 36M.

It should also be emphasised that Raman mapping in Part 1 was performed mainly on selected air-side regions. The present results therefore demonstrate lateral heterogeneity at the film surface, but they do not yet establish the full through-thickness distribution of the salt domains. Cross-sectional Raman depth profiling and/or SEM-EDS will be required to assess depth localisation in the next stage.

#### 2.3.4. Confocal Raman Depth Profiling of Films 26M and 36M

**Figure 8:**
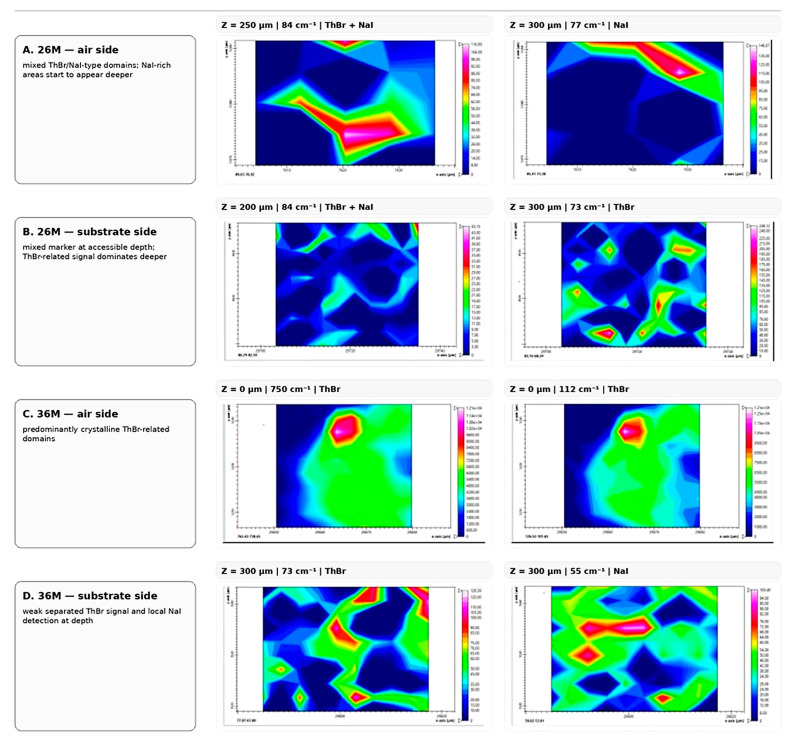
Representative confocal Raman depth profiling of films 26M and 36M from the air side and substrate side at selected depths. The figure should highlight the spectral markers used to distinguish mixed ThBr/NaI-type domains in 26M from predominantly separated ThBr- and NaI-containing domains in 36M. The complete depth-profiling map sets should be presented in Supplementary Figures S10 and S11.

Confocal Raman depth profiling was performed to examine the distribution of salt-containing domains across the accessible near-surface regions from both the air side and the substrate side of films 26M and 36M. Because the spectra became weak and noisy at depths beyond approximately 500 μm, the measurements were collected from both film surfaces and should not be interpreted as a complete full-thickness cross-section. Three representative regions on each surface showed the same general trend. A representative summary is shown in Figure 8, while the full depth-profiling maps should be placed in Supplementary Figures S10 and S11.

In film 26M, the air-side profiles were consistent with a higher fraction of mixed ThBr/NaI-type domains within the outer approximately 300 μm, whereas at greater accessible depths, separate NaI-rich regions began to appear in addition to these mixed domains. From the substrate side, both ThBr- and NaI-related bands were detected near the surface, while deeper accessible regions were dominated mainly by ThBr-related bands.

In film 36M, the depth profiles were more consistent with predominantly separated ThBr- and NaI-containing domains. The air side showed bands attributable mainly to crystalline ThBr, whereas from the substrate side, only weak salt-related bands remained at greater accessible depths, with NaI becoming detectable only locally.

Overall, the confocal depth-profiling results support the same trend as the surface Raman maps: 26M contains a higher fraction of mixed salt-containing domains, whereas 36M is characterised mainly by more isolated ThBr and NaI crystallites. These data extend the surface observations into the accessible near-surface layers and strengthen the structural interpretation of the two films.

### 2.4. Release of ThBr and NaI from Films 26M and 36M

**Figure 9:**
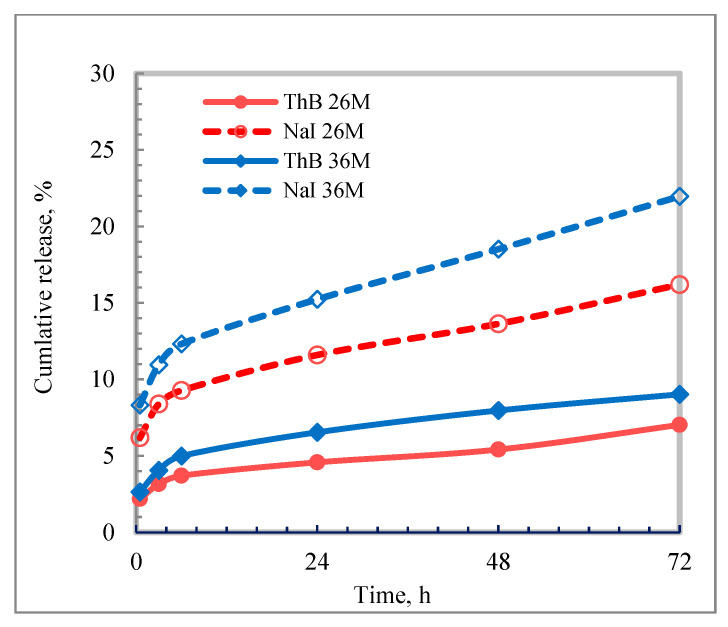
Cumulative release of ThBr and NaI from matrix films 26M and 36M (mean ± SD, *n* = 3).

The cumulative release profiles of ThBr and NaI from films 26M and 36M are shown in Figure 9. After 72 h, release remained low for both salts (7 and 9% for ThBr; 16 and 22% for NaI from films 26M and 36M, respectively), while swelling was only 1–1.5%. These data confirm limited release from hydrophobic PDMS matrices. In the context of the planned application-oriented Part 2, such values should presently be interpreted as proof-of-concept matrix behaviour rather than evidence of practical transdermal performance.

The factors governing the release of water-soluble compounds from hydrophobic polymer matrices into aqueous media have been discussed in classical studies on water uptake, osmotic effects, and salt release from silicone or hydrophobic polymer matrices [23,24,25,26]. In general, water may penetrate the polymer network through microdefects formed during curing and/or through the silicone phase acting as a partially permeable membrane. Within salt-containing domains, ThBr and NaI crystallites can dissolve and form locally concentrated electrolyte solutions. The dissolved salts then diffuse into the external aqueous medium through available interfacial channels, microcracks, and connected diffusion pathways. Local osmotic pressure at the cavity–matrix interface may further promote water ingress and help maintain the concentration gradient; in extreme cases, it may also contribute to additional microdefect formation. Thus, the overall release profile is controlled by the combined effects of water penetration, dissolution of crystalline salt domains, diffusion through the hydrophobic network, and possible osmotic phenomena.

In films 26M and 36M, the low overall release can be explained by three formulation-specific factors. First, the PDMS–TEOS network is highly hydrophobic and exhibits only minimal swelling, approximately 1–1.5%, which severely limits water uptake. Second, Raman spectroscopy, Raman mapping, confocal Raman depth profiling, and XRD show that ThBr and NaI are not molecularly dispersed in the matrix but are present as discrete crystalline domains, including individual ThBr/NaI crystallites and mixed salt-containing domains. Third, only salt domains accessible to water through the weakly swelling matrix, interfacial channels, or microdefects can contribute substantially to release over 72 h. Consequently, although ThBr and NaI are highly water-soluble, a large fraction of the incorporated salts probably remain trapped inside the hydrophobic matrix during the experiment.

The different release extents of ThBr and NaI should also be interpreted cautiously. NaI was released in larger amounts than ThBr, which may reflect greater accessibility of NaI-rich domains and/or a stronger contribution of local osmotic effects. The structural differences between 26M and 36M, namely a higher fraction of mixed ThBr/NaI-containing domains in 26M and more separated salt crystallites in 36M, may contribute to the observed release behaviour. However, this relationship should be regarded as a plausible structure–release correlation rather than direct causal proof. Control films containing only ThBr or only NaI, as well as more complete cross-sectional compositional analyses, will be needed to test this interpretation directly.

The amount and kinetics of the released salts were analysed using standard mathematical models of drug release [27,28]. Table 1 summarises the kinetic parameters obtained by fitting the experimental curves to zero-order, Higuchi, and Korsmeyer–Peppas models.

The mathematical fits are useful for comparative description of films 26M and 36M, but their mechanistic interpretation must remain cautious. The released fraction remained far below the range typically used for confident application of the Korsmeyer–Peppas model, and the apparent linearity over a narrow interval does not establish a true zero-order mechanism.

Accordingly, the present results are best interpreted as being consistent with diffusion-limited release from hydrophobic, weakly swelling matrices, with possible contributions from local dissolution and osmotic effects in salt-containing domains. The modelling supports comparison between formulations but does not by itself prove a definitive release mechanism.

## 3. Materials and Methods

### 3.1. Materials

#### 3.1.1. Thiamine Bromide (ThBr)

Thiamine bromide hydrobromide (3-[(4-amino-2-methylpyrimidin-5-yl)methyl]-5-(2-hydroxyethyl)-4-methyl-1,3-thiazol-3-ium bromide hydrobromide, ThBr) was synthesised at the Scientific Technological Centre of Organic and Pharmaceutical Chemistry, NAS RA (Yerevan, Armenia).

#### 3.1.2. Sodium Iodide (NaI)

Sodium iodide (NaI, ≥99.5%) was purchased from VWR Chemicals (VWR International SAS, Rosny-sous-Bois, France) and used as received.

#### 3.1.3. TIODIN Injectable Solution

TIODIN is a combined injectable product containing thiamine bromide (ThBr) and sodium iodide (NaI) in aqueous solution. One ampoule contains 0.063 g of ThBr and 0.05 g of NaI dissolved in 5 mL of distilled water (manufacturer: Scientific Technological Centre of Organic and Pharmaceutical Chemistry, NAS RA, Yerevan, Armenia).

#### 3.1.4. TEOS and Catalyst

Tetraethoxysilane (TEOS, 99%) and stannous 2-ethylhexanoate (tin(II) 2-ethylhexanoate, Sn(Oct)_2_, 92.5–100%) were purchased from Sigma-Aldrich and used as received.

#### 3.1.5. Hydroxyl-Terminated PDMS

Hydroxyl-terminated polydimethylsiloxanes (PDMS–OH) with different viscosities and molecular weights were obtained from Gelest:

- DMS-S31: viscosity 1000 cSt, M_n_ ≈ 26,000 g·mol^−1^ (26k);
- DMS-S32: viscosity 1800–2200 cSt, M_n_ ≈ 36,000 g·mol^−1^ (36k);
- DMS-S323: viscosity 3500 cSt, M_n_ ≈ 43,500 g·mol^−1^ (43k).

### 3.2. Methods

#### 3.2.1. UV–Vis Spectroscopy, FTIR, Raman Spectroscopy, and XRD

The release of ThBr and NaI into 0.9% (*w*/*w*) aqueous NaCl solution (physiological saline) was monitored by UV–Vis spectroscopy using a Cary 100 UV–Vis spectrophotometer (Agilent Technologies, Santa Clara, CA, USA). Calibration curves were obtained separately for ThBr, NaI, and their mixtures in distilled water and in 0.9% NaCl solution (see the Supplementary Materials, Figure S1).

In this first part of this study, 0.9% NaCl solution was selected as a simple comparative release medium and because the UV–Vis calibration procedure was established in this medium (see the Supplementary Materials). It was not intended to reproduce skin permeation conditions; such an assessment requires dedicated in vitro permeation testing (IVPT), typically in diffusion-cell systems using skin or other relevant barrier membranes [29].

FTIR spectra of NaI, ThBr, and the ThBr/NaI powder mixture were recorded on an Avatar Nicolet FTIR spectrometer (Thermo Nicolet, Hillsboro, OR, USA) using KBr pellets. Spectra were collected in the range 400–4000 cm^−1^ with a resolution of 4 cm^−1^ and averaged over 32 scans.

Raman measurements were performed using a Bruker SENTERRA II Raman microscope (Bruker Optics GmbH & Co. KG, Ettlingen, Germany). A diode laser with an excitation wavelength of 785 nm and a power of 50 mW was used in combination with an OLYMPUS 50× objective lens (N = 0.65) (Olympus Corporation, Tokyo (Hachioji), Japan). Raman spectra were recorded in the 50–3200 cm^−1^ range. For Raman mapping, selected regions of the film surfaces of 90 × 60 µm were scanned with a step size of 2 µm in both the *x* and *y* directions, resulting in approximately 2500 spectra per map. The Raman maps were processed and analysed using the OPUS software (v. 8.7, Bruker Optics GmbH & Co. KG, Ettlingen, Germany).

Confocal Raman depth profiling of films 26M and 36M was performed from both the air side and the substrate side by collecting Raman maps at successive focal depths. Because signal intensity decreased substantially beyond approximately 500 μm, the analysis was restricted to accessible near-surface layers. For each surface, three representative regions were examined in a faster acquisition mode (100 spectra per map) and selected depths were compared using diagnostic low-frequency and thiamine-related Raman bands.

X-ray diffraction patterns of the powders (NaI, ThBr, and ThBr/NaI-P) and of the PDMS-based films were recorded on an EMPYREAN diffractometer (PANalytical B.V., Almelo, Netherlands) operated at 45 kV and 40 mA using Cu Kα radiation (λ = 1.5406 Å). Data were collected in θ–2θ geometry. The diffractometer settings were as follows: 2θ range 5–90°, step size 0.013° and counting time per step 8.67 s.

#### 3.2.2. Preparation of PDMS Films (PDMS-p, 26M, 36M)

The TIODIN-containing composite films were prepared using crosslinked elastomeric matrices formed by the reaction of PDMS–OH with TEOS in the presence of Sn(Oct)_2_ as a catalyst. ThBr and NaI were used as powders in a mass ratio of 1.2/1.0 (ThBr/NaI), corresponding to their ratio in the injectable TIODIN solution for intramuscular administration. The weighed amounts of ThBr and NaI were added to a pre-weighed portion of PDMS–OH (26k or 36k) in a glass beaker and mixed until a visually homogeneous fine dispersion was obtained. TEOS was then added, and the mixture was stirred for about 10 min, followed by the addition of Sn(Oct)_2_. Stirring was continued until a noticeable increase in viscosity indicated the onset of crosslinking. The viscous mixture was poured into plastic Petri dishes and left at room temperature (23–25 °C) to cure. The time required for film formation (crosslinking/curing) was recorded. After 2 days at room temperature, the films were placed in a thermostatic oven at 37 ± 0.1 °C and kept for an additional 24 h.

Attempts to prepare a film using the highest-viscosity PDMS–OH (43k) were unsuccessful: due to the high viscosity, PDMS–OH mixed poorly with ThBr and NaI, resulting in a highly inhomogeneous, rough film. Therefore, films with 43k PDMS–OH were not studied further. Reference PDMS films without ThBr and NaI salts (PDMS-p) were prepared by the same procedure.

The compositions of the films are summarised in Table 2.

#### 3.2.3. Release

Release of ThBr and NaI into 0.9% NaCl solution in vitro was determined as follows. From each film, three fragments with a weight of 0.05–0.10 g were cut and used as parallel samples (*n* = 3). Throughout the experiment, the tubes were kept in a thermostatic bath at 37 ± 0.1 °C. At predetermined time intervals, the release medium was analysed by UV spectroscopy and the film fragments were removed, gently blotted with filter paper, weighed, and then returned to fresh portions of 0.9% NaCl solution. The cumulative release data shown in Figure 9 should be presented as mean values with the corresponding variability measure.

Swelling (%) = [(W – W0)/W0] × 100%

where W_0_ is the initial weight of the dry sample and W is the weight of the swollen sample at a given time.

#### 3.2.4. Preparation of ThBr/NaI Mixed Crystals (ThBr/NaI-P)

Mixed crystals of ThBr with NaI (ThBr/NaI-P) were obtained from the TIODIN injectable solution. The contents of one ampoule (5 mL aqueous solution containing 0.063 g ThBr and 0.05 g NaI) were transferred to a glass beaker and left in the dark at room temperature (22–24 °C) until most of the water had evaporated. Residual moisture was removed in a vacuum oven at 35 ± 1 °C. The resulting crystalline powder was characterised by FTIR, Raman spectroscopy, and XRD. These data were used to identify possible interactions between ThBr and NaI occurring during the formation of composite polysiloxane films (26M and 36M) containing ThBr and NaI.

#### 3.2.5. Film Thickness Measurements

Film thickness was measured using a mechanical micrometre (Matrix Precision Co., Ltd., Hsinchu City, Taiwan). For each film, measurements were performed at several points across the film surface, and the mean value and standard deviation were calculated.

## 4. Conclusions

The present study shows that films 26M and 36M are hydrophobic PDMS-based matrices in which ThBr and NaI occur as individual crystallites and/or mixed salt-containing domains. The combined FTIR, Raman mapping, confocal Raman depth-profiling, and XRD data support a higher fraction of mixed domains in 26M and a predominance of more isolated crystallites in 36M; however, the detailed phase assignments remain tentative and should be interpreted cautiously.

Release of both salts into physiological saline remained low over 72 h, and film swelling was minimal. The confocal depth-profiling results extended the surface observations into the accessible near-surface layers, but they did not provide a complete full-thickness compositional map. Taken together, these findings identify a clear limitation of the current formulations: in their present form, they should be regarded as proof-of-concept silicone matrices rather than validated transdermal patch systems. In addition, Part 1 does not include a separate adhesive layer, tack/adhesion testing, skin permeation studies, or therapeutic evaluation.

The main value of Part 1 is therefore mechanistic and preformulation-oriented. It identifies matrix composition, salt-domain microstructure, accessible depth distribution, and accessibility to water as key variables for further optimisation. The planned Part 2 is intended to address formulation optimisation more directly, including single-salt control films; possible hydrophilic modifiers; adhesive/handling studies; dedicated in vitro permeation testing; and, where needed, more extensive cross-sectional compositional characterisation.

## Figures and Tables

**Table 1:** Kinetic parameters for zero-order, Higuchi, and Korsmeyer–Peppas models.

Release Models	26M		36M	
	ThBr	NaI	ThBr	NaI
	R^2^	R^2^	R^2^	R^2^
Zero-order	0.981	0.988	0.955	0.991
Higuchi	0.956	0.989	0.994	0.988
Korsmeyer–Peppas	0.944	0.969	0.993	0.966
Korsmeyer–Peppas (*n*)	0.224	0.196	0.241	0.206

**Table 2:** Composition of PDMS-p, 26M, and 36M films.

Components (in the Initial Mixture), w.%	PDMS-p	26M	36M
ThBr	-	3.1	3.1
NaI	-	2.5	2.5
PDMSOH (26k, 36k)	95.4	88.8	90.1
TEOS	4.3	5.3	4.0
Sn(oct)2	0.3	0.3	0.3
Film thickness, mm (mean ± standard deviation)	1.0 ± 0.1	1.6 ± 0.28	1.65 ± 0.16

## Data Availability

The original contributions presented in this study are included in this article/its Supplementary Materials. Further inquiries can be directed to the corresponding authors.

## Supplementary Materials

The following supporting information can be downloaded at https://www.mdpi.com/article/10.3390/molecules31101588/s1, Figure S1: UV—Vis spectra of (a) NaI in water and 0.9% NaCl, (b) ThBr in 0.9% NaCl (4 mg/100 g solution), and (c) mixtures of ThBr and NaI in 0.9% NaCl (2.5 mg/100 g solution).; Figure S2: Raman spectra of (a) ThBr, (b) NaI and (c) their mixture ThBr/NaI-P (powder); Figure S3: FTIR spectra of ThBr and ThBr/NaI-P (powder); Figure S4: Raman spectra of the PDMS-P film; Figure S5: Raman spectra of film 26M (an example); Figure S6: Raman spectra of film 36M (an example); Figure S7: Raman mapping of film 26M, map 1 (air side); Figure S8: Raman mapping of film 36M, map 1 (air side); Figure S9: Raman mapping of film 36M, map 3 (bands at 59 and 112 cm^−1^); Figure S10: Complete confocal Raman depth-profiling map set of film 26M recorded from the air side and substrate side at selected depths. The maps show the spatial distribution of Raman bands assigned to ThBr-related, NaI-related and associated ThBr/NaI-type domains; Figure S11: Complete confocal Raman depth-profiling map set of film 36M recorded from the air side and substrate side at selected depths. The maps show Raman markers associated with pre-dominantly separated ThBr- and NaI-containing domains; Table S1:Experimental FTIR and Raman wavenumbers (cm^−1^) and band assignments for ThBr, NaI and ThBr/NaI-P; Table S2: XRD comparison for NaI, ThBr and ThBr/NaI-P; Table S2a: Reference Peaks (ThBr); Table S2b: Reference Peaks (NaI); Table S3. Main Raman bands of the PDMS film and their assignments; Table S4: XRD peak assignments for film 26M: a—air side, b—substrate side; Table S5. XRD peak assignments for film 36M: a—air side, b—substrate side.

## Abbreviations

The following abbreviations are used in this manuscript:

API	Active Pharmaceutical Ingredient
AS	Air Side
cP	Centipoise
NAS RA	National Academy of Sciences of the Republic of Armenia
NaCl	Sodium Chloride
*n*	Diffusional Exponent
NaI	Sodium Iodide
NaI,Br	Mixed Sodium Iodide/Bromide
PD	Parkinson’s Disease
PDMS	Polydimethylsiloxane
PDMS--OH	Hydroxyl-Terminated Polydimethylsiloxane
PDMS-p	Pure PDMS Film (Reference)
PSA	Pressure-Sensitive Adhesive
R^2^	Coefficient of Determination
R5	Thiazole Ring
R6	Pyrimidine Ring
R5 breath	Thiazole Ring Breathing Mode
R6 breath	Pyrimidine Ring Breathing Mode
SnOct	Stannous 2-Ethylhexanoate (Tin(II) 2-Ethylhexanoate)
SS	Substrate Side
TDDS	Transdermal Drug Delivery Systems
TEOS	Tetraethoxysilane
ThBr	Thiamine Hydrobromide
ThBrNaI-P	ThBrNaI Powder (Mixed Crystals from TIODIN Solution)
ThCl	Thiamine Hydrochloride
ThI	Thiamine Iodide
ThI,Br	Mixed Thiamine Iodide/Bromide
TIODIN	Thiamine Bromide + Sodium Iodide Injectable Solution
XRD	X-ray Diffraction
