# Supplementary material for: Development of PDMS Films Containing Thiamine Bromide and Sodium Iodide: Part 1—Matrix Characterisation and In Vitro Release
Source: Molecules. 2026 May 9;31(10):1588. doi: 10.3390/molecules31101588 (PMC13209605; doi:10.3390/molecules31101588)
Supplement: Supplementary file 1 [file molecules-31-01588-s001.zip › molecules-4262088-supplementary.pdf]

molecules

Supplementary Materials

Development of PDMS Films Containing Thiamine Bromide and Sodium Iodide: Part 1—Matrix Characterisation and In Vitro Release

Zoya Farmazyan [1,*], Nelli Avagyan [1], Vigen Topuzyan [1], Emma Arakelova [2], Stepan Grigoryan [1], Mari Atabekyan [1], Susanna Grigoryan [2], Karen Khachatryan [3] and Gohar Khachatryan [4,*]

[1] The Scientific Technological Centre of Organic and Pharmaceutical Chemistry NAS RA, 26, Azatutyan Str., Yerevan 0014, Armenia; nelli.avagyan80@gmail.com (N.A.); vtop@web.am (V.T.); grigstepan@yahoo.com (S.G.); atmari@yandex.ru (M.A.)
[2] Laboratory of X-Ray Structural Studies, National Polytechnic University of Armenia, 105, Teryan Str, Yerevan 0009, Armenia; emma_arakelova@yahoo.com (E.A.); susanna.grigorian@mail.ru (S.G.)
[3] Laboratory of Nanotechnology and Nanomaterials, Faculty of Food Technology, University of Agriculture in Krakow, Al. Mickiewicza 21, 31-120 Krakow, Poland; karen.khachatryan@urk.edu.pl
[4] Department of Food Analysis and Quality Assessment, Faculty of Food Technology, University of Agriculture in Krakow, Al. Mickiewicza 21, 31-120 Krakow, Poland
* Correspondence: zoefa2000@yahoo.com (Z.F.); gohar.khachatryan@urk.edu.pl (G.K.)

Supplementary Materials

1. UV–Vis spectroscopy and calibration

The release of ThBr and NaI from films into 0.9% (w/w) aqueous NaCl solution (physiological saline) was monitored by UV–Vis spectroscopy using a Cary 100 UV–Vis spectrophotometer (Agilent Technologies). Calibration curves were obtained separately for ThBr, NaI and their mixtures in distilled water and in 0.9% NaCl solution.

Aqueous NaCl solutions at concentrations ≤ 0.9% show an absorption band with a maximum at 197–198 nm. The UV spectra of NaI in water and in 0.9% NaCl are identical, with a characteristic absorption maximum at 226 nm (Fig. S1a). The UV spectra of ThBr in water and in 0.9% NaCl are also identical. For ThBr solutions at concentrations ≤ 5 mg per 100 g of solution, two absorption bands are observed at 262 and 237 nm (Fig. S1b).

In the UV spectra of NaI and ThBr mixtures (in saline), two absorption maxima are observed at 226 nm and 262–265 nm (Fig. S1c), which were used for the quantitative determination of the released NaI and ThBr, respectively.

 https://doi.org/10.3390/molecules31101588

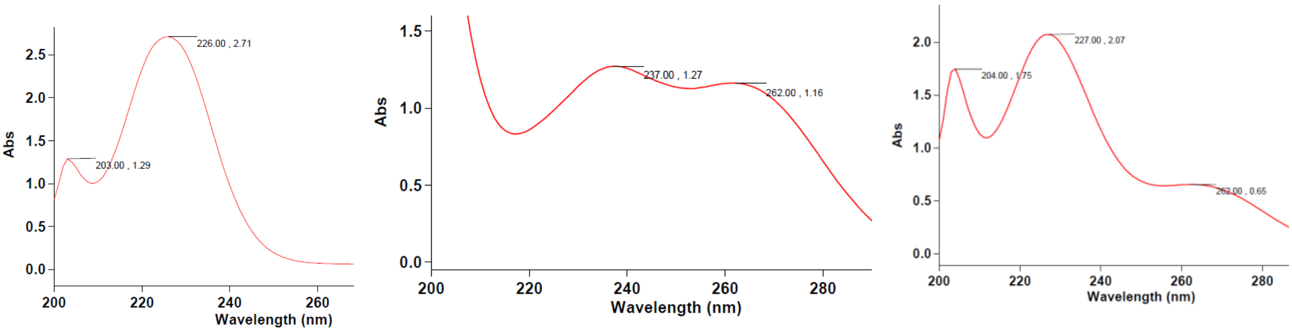

a b c

Figure S1. UV–Vis spectra of (a) NaI in water and 0.9% NaCl, (b) ThBr in 0.9% NaCl (4 mg/100 g solution), and (c) mixtures of ThBr and NaI in 0.9% NaCl (2.5 mg/100 g solution).

2. Raman and FTIR spectra of NaI, ThBr and ThBr/NaI

Raman study was carried out using a Bruker SENTERRA II Raman spectrometer. The laser wavelength was 785 nm with a power of 50 mW, and an OLYMPUS 50x objective was selected. Raman spectra were recorded in the range of 50–3200 cm^{-1}. For mapping, areas of 90×60 μm were selected with a 2 μm step, resulting in the collection of approximately 2500 spectra. The mapping results were processed using the OPUS software.

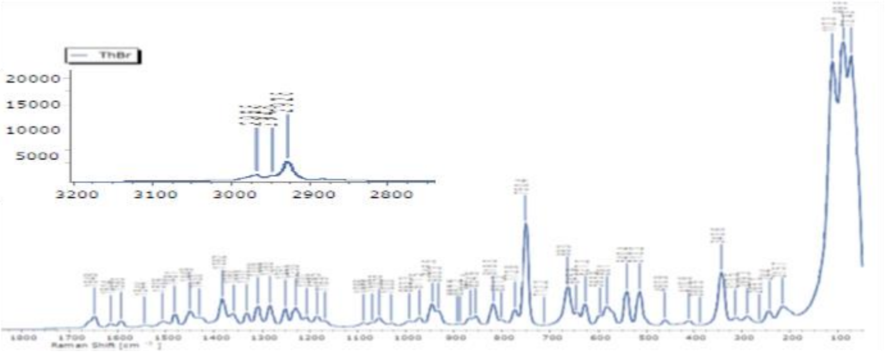

a

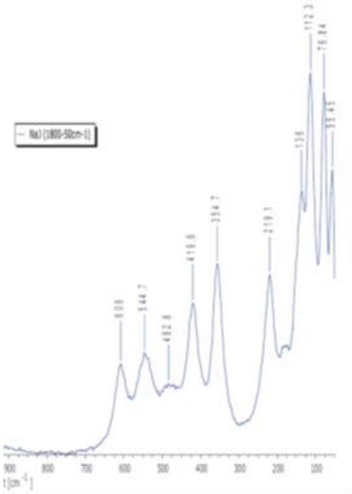

b

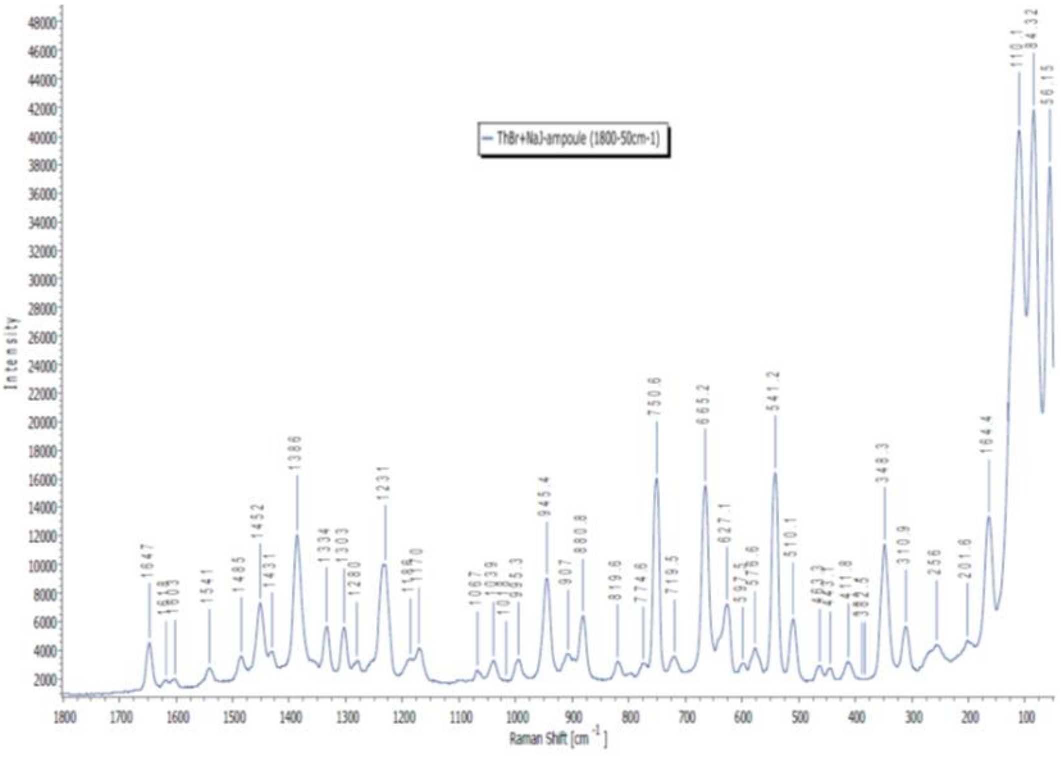

c

Figure S2. Raman spectra of (a) ThBr, (b) NaI and (c) their mixture ThBr/NaI-P (powder).

FTIR spectra of ThBr and the ThBr /NaI- P were recorded on an Avatar Nicolet FTIR spectrometer (Thermo Nicolet, USA) using KBr pellets. Spectra were collected in the range 400–4000 cm^{-1} with a resolution of 4 cm^{-1} and averaged over 32 scans.

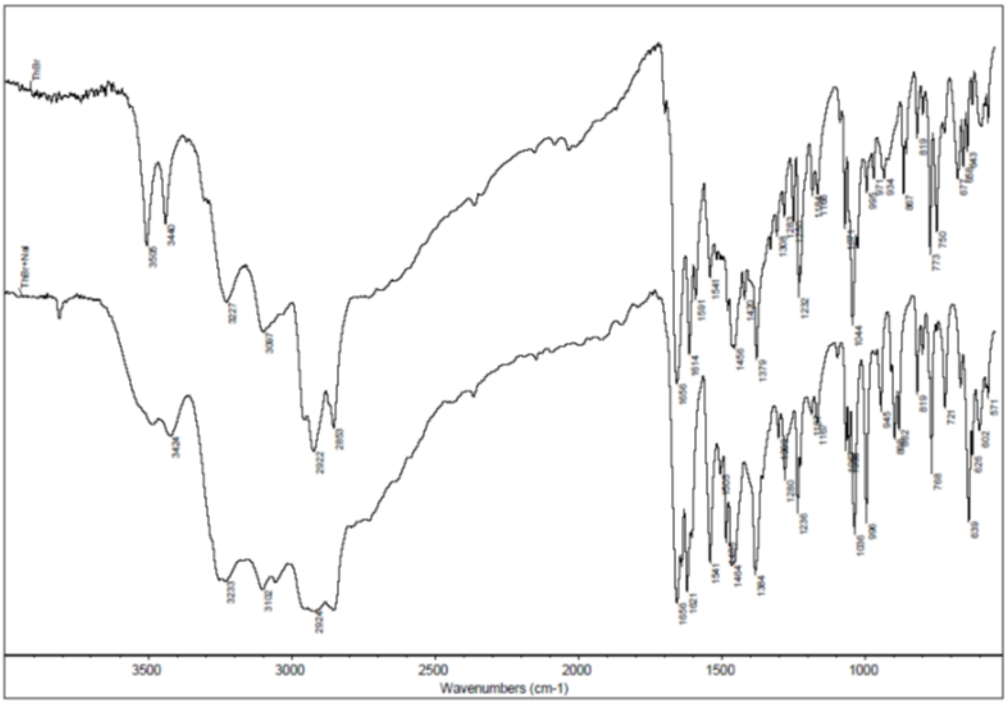

Figure S3. FTIR spectra of ThBr and ThBr/NaI-P (powder).

Table S1. Experimental FTIR and Raman wavenumbers (cm^{-1}) and band assignments for ThBr, NaI and ThBr/NaI-P.

**FTIR cm^{-1}**			**Raman shift cm^{-1}**	
Assignments	ThBr	ThBr +NaI (powder)	ThBr	ThBr +NaI (powder)
Str OH<br>str NH(NH$_2$)	3505<br>3440	Merged into a wide band with max at 3500 cm^{-1}	-	-
NH(NH$_2$) +N$_1$H bend	1656	1656	1648	1648
str CO<br>str CO+strR6	1044<br>1071<br>-	1036<br>1058<br>1067	1030,1055<br>1070,1088	1039-40<br>1067
R6 breath	750	768	750.4	750.6
R5 breath	971, 934	945	945.5	945.5
CS str	867, 934<br>971	882, 897	867.1, 854.6	880.8
o.p.def R$_5$+CS	658	667	663	665.2
o.p. def NH(NH$_2$)	571	571	540.4, 581	541.2, 576.6
o.p. defR$_5$<br>o.p. defR$_6$	-	-	343.6<br>313.5	348.3<br>310.9
def C5C6 Nd<br>def OH<br>o.p. def C7SC9	-	-	289.3<br>244<br>215.7	disaapered<br>256,201<br>disaapered
<200 cm^{-1} **lattice phonon vibrations,** ThBr	-	-	112.3 89.87<br>73.43	201.6, 164.4, 110.1, 84.32, 56.13
<200 cm^{-1} **lattice phonon vibrations,** NaI	-	-	112.3, 76.84 (77-78) and 55.45 (55-56)	

Abbreviations: str – stretching, def- deformation, o.p.- out of plane

3. XRD phase analysis of ThBr, NaI and ThBr/NaI-P

XRD diffraction patterns of NaI, ThBr **, ThBr/NaI-P** and PDMS-based films were recorded on an EMPYREAN diffractometer (PANalytical, The Netherlands) operated at 45 kV and 40 mA using Cu Kα radiation (λ = 1.5406 Å). Data were collected in θ–2θ geometry. The diffractometer settings are 2θ range 5-90^0, step size 0.013^0 and counting time per step 8.67s).

Table S2. XRD comparison for NaI, ThBr and ThBr/NaI-P.

**ThBr /NaI-P 2θ (°)/ d (Å)**	**Closest 2θ (NaI / ThBr)**	**Assignment**
26.37/3.37	26.25/3.4 NaI<br>26.51/3.36ThBr	Consistent with ThBr and NaI
30.35/2.94	29.93/2.99 NaI<br>29.92/3.24 ThBr	Consistent with ThBr and NaI
43.34/2.08	42.71/1.93 NaI<br>41.31/2.18 ThBr	Na(I,Br) or Th(I,Br) (±H2O)
51.20/1.78	52.25/1.72 NaI	NaI-like
53.58/1.71	53.22/1.5 NaI	NaI-like
62.63/1.48	61.80/1.45 NaI	NaI-like
69.04/1.36	70.25/1.32 NaI	NaI-like
70.91/1.32	70.25/1.32 NaI	NaI-like
71.27/1.32	71.63/1.31 NaI	NaI-like
78.87/1.21	-	New phase
84.61/1.14	-	New phase

https://doi.org/10.3390/molecules31101588

Table S2a. Reference Peaks (ThBr)

ThBr (Thiamine Bromide)	
2θ (°)	d (Å)
8.29	10.66
17.00	5.21
18.66	4.75
26.51	3.36
26.63	3.35
27.26	3.27
29.92	3.24
30.92	2.98
32.60	2.89
33.69	2.80
41.31	2.18

Table S2b. Reference Peaks (NaI)

NaI	
2θ (°)	d (Å)
15.35	5.77
17.30	5.13
18.33	4.84
20.89	4.25
22.22	4.00
24.90	3.58
25.42	3.50
25.63	3.48
26.25	3.40
26.66	3.34
27.63	3.23
28.09	3.18
28.41	3.14
29.93	2.99
32.97	2.71
33.71	2.54

https://doi.org/10.3390/molecules31101588

35.31	2.36
38.11	2.23
40.41	2.19
41.17	2.11
42.71	1.93
47.05	1.86
48.79	1.84
49.37	1.75
52.25	1.72
53.22	1.50
61.80	1.45
64.22	1.34
70.25	1.32
71.63	1.31

3.1. Key Hydrolysis–Condensation Reactions between PDMS–OH and Tetraethoxysilane (TEOS)

The key reactions involved in the synthesis of crosslinked polydimethylsiloxane (PDMS) via condensation between PDMS–OH and tetraethoxysilane (TEOS), catalyzed by stannous octoate ($Sn(Oct)_2$), have been investigated in detail elsewhere [1-4] and are summarized below:

1. Hydrolysis of TEOS:

$$Si(OC_2H_5)_4 + x\ H_2O \rightleftharpoons Si(OC_2H_5)_{4-x}(OH)_x + x\ C_2H_5OH$$

2. Condensation and Crosslinking with PDMS–OH (water or ethanol elimination):

a) Silanol Condensation Pathway:

$Si(OH)_4 + HO–PDMS–OH \rightarrow Si(OH)_3–O–[PDMS]–OH + H_2O$

b) Alkoxy Condensation Pathway:

$Si(OC_2H_5)_4 + 4HO–PDMS–OH \rightarrow Si–[O–PDMS]_4 + 4C_2H_5OH$.

3. Self-condensation of TEOS (formation of SiO_2 network):

a) Silanol Condensation:

$\equiv Si–OH + HO–Si\equiv \rightarrow \equiv Si–O–Si\equiv + H_2O$

b) Alkoxy Condensation:

$\equiv Si–OR + HO–Si\equiv \rightarrow \equiv Si–O–Si\equiv + ROH$

4. Raman spectra and XRD of the films PDMS-P, 26M and 36M

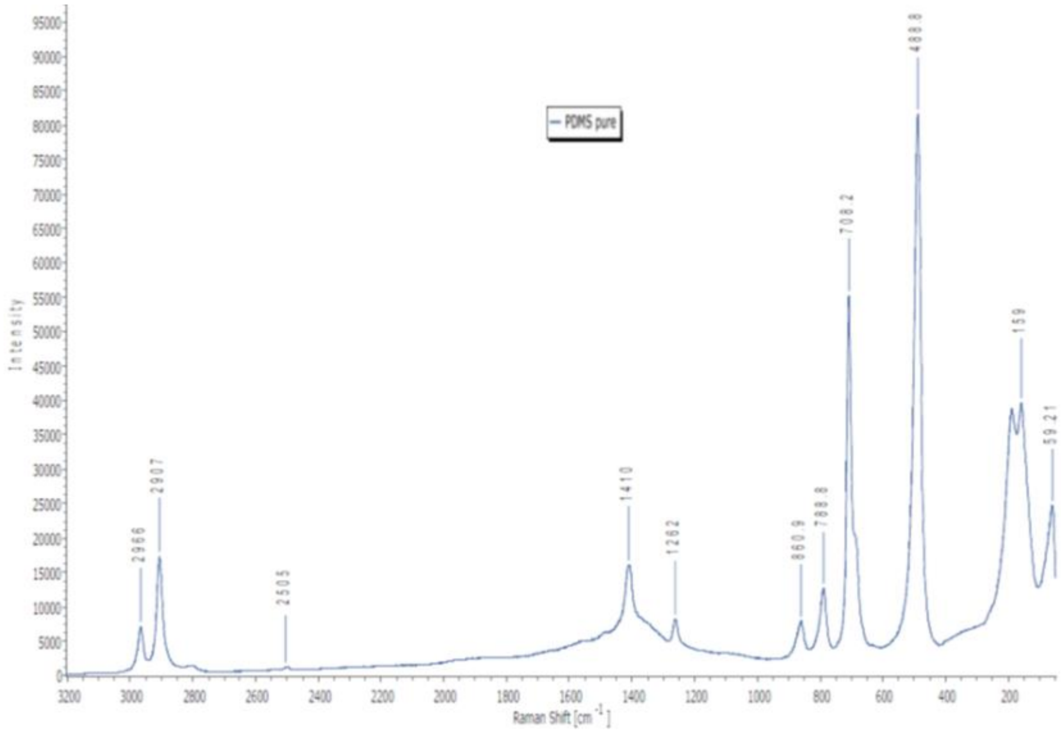

Figure S4. Raman spectra of the PDMS-P film

Table S3. Main Raman bands of the PDMS film and their assignments.

**Wave-number, cm^{-1}**	**Assignment**
2907, 2966	CH_3 asymmetric and symmetric stretching
1410	CH_3 asymmetric defor-mation
1262	CH_3 symmetric defor-mation
861	CH_3 rocking
789	C-Si-C asymmetric stretching
709	C-Si-C symmetric stretching
489	**Si–O–Si** symmetric stretching
190	C-Si-C wagging
159	C-Si-C twisting
59.21	**Si–O–Si** scissors

https://doi.org/10.3390/molecules31101588

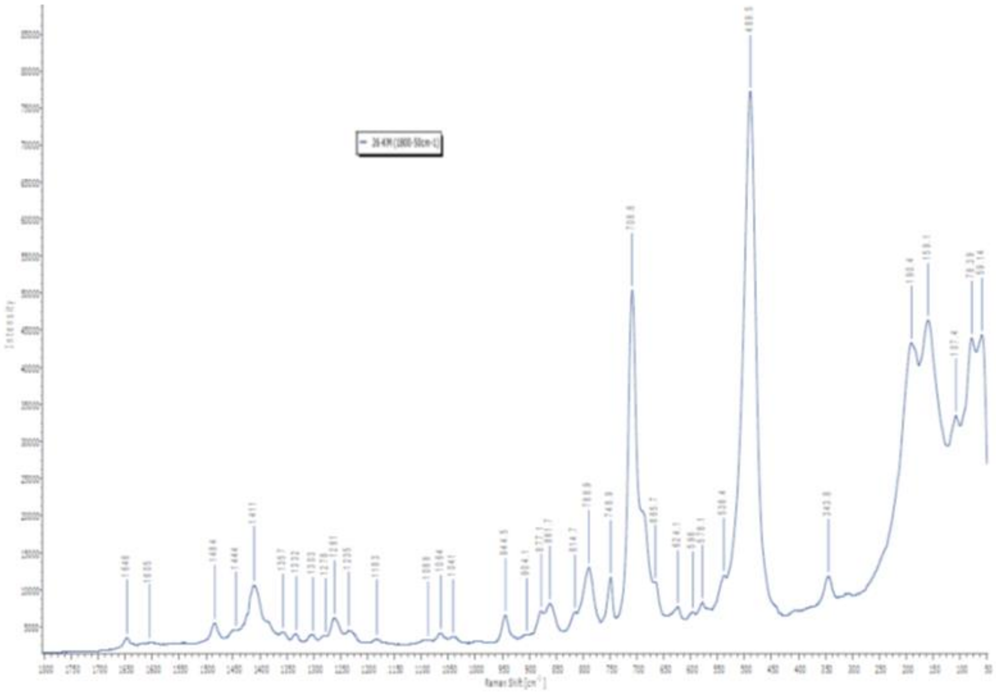

Figure S5. Raman spectra of film 26M (an example).

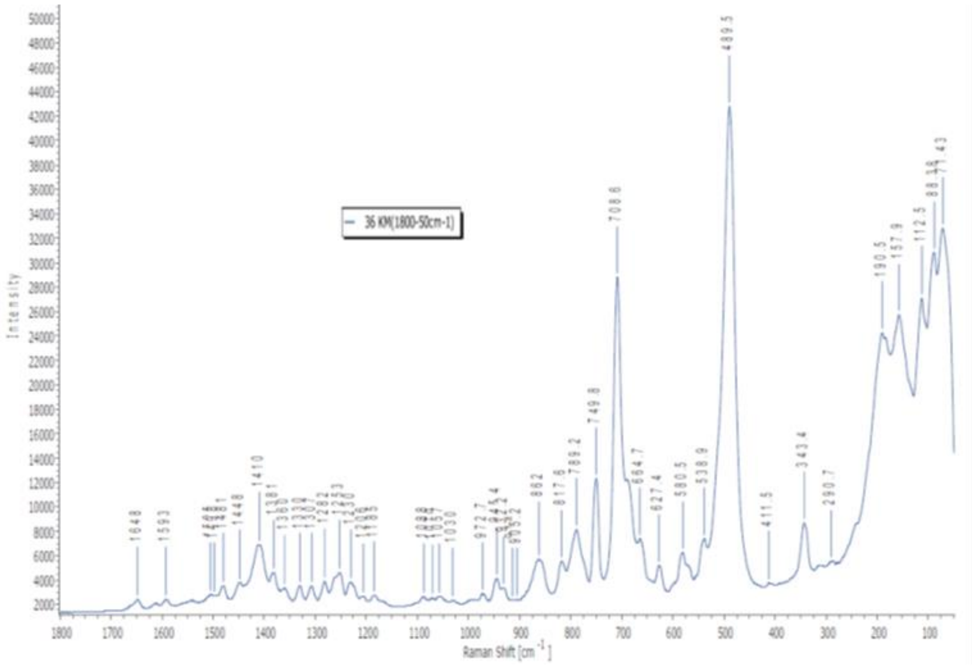

Figure S6. Raman spectra of film 36M (an example).

Table S4. XRD peak assignments for film 26M: a – air side, b – substrate side.

26M- a, air-side.

**2θ (°) / d (Å)**	**Closest NaI**	**Closest ThBr**	**Assignment**
26.89/3.32	26.66/3.34	26.63/3.35	ThBr and NaI, mix or overlap
29.84/2.99	29.93/2.99	29.92/3.24	ThBr and NaI, mix or overlap
31.18/2.87	29.93/2.99	30.92/2.98	ThBr- like
32.95/2.72	32.97/2.71	32.60/2.89	NaI
36.41/2.47	35.31/2.36	33.69/2.8	New
41.62/2.17	41.17/2.11	41.31/2.18	ThBr- like
43.45/2.08	42.71/1.93	41.31/ 2.18	New

26M- b, substrate-side

**2θ (°) / d (Å)**	**Closest NaI**	**Closest ThBr**	**Assignment**
11.73/7.54	15.35/5.77	8.29/10.66	Th(I,Br) (±H2O)
26.58/3.35	26.66/3.34	26.63/3.35	ThBr and NaI, mix or overlap
38.45/2.34	38.11/2.23	41.31/2.18	NaI or Na(I,Br)

https://doi.org/10.3390/molecules31101588

Table S5. XRD peak assignments for film 36M: a – air side, b – substrate side.

36M- a, air-side

2θ (°) / d (Å)	Closest NaI	Closest ThBr	Assignment
7.80/10	15.35/5.77	8.29/10.66	ThBr (shifted)
17.10/5.18	17.30/5.13	17.00/5.2	ThBr-like
26.65	26.66	26.63	ThBr and NaI, mix or overlap
27.16/3.28	27.63/3.23	27.26/3.27	ThBr
28.99/3.08	28.41/3.14	29.92/3.24	New
31.06/2.88	29.93/2.99	30.92/2.98	ThBr-like
34.72/2.58	35.31/2.36	33.69/2.8	New
41.38/2.18	41.17/2.11	41.31/2.18	ThBr-like
47.72/1.91	47.05/1.86	41.31/2.18	NaI - like

36M- b, substrate-side

2θ (°) / d (Å	Closest NaI	Closest ThBr	Assignment
26.75/3.33	26.66/3.34	26.63/3.35	ThBr and NaI, mix or overlap
41.45/2.18	41.17/2.11	41.31/2.18	ThBr-like

https://doi.org/10.3390/molecules31101588

5. Raman mapping of films 26M and 36M

**26M map1**	
84 ThBr/ NaI 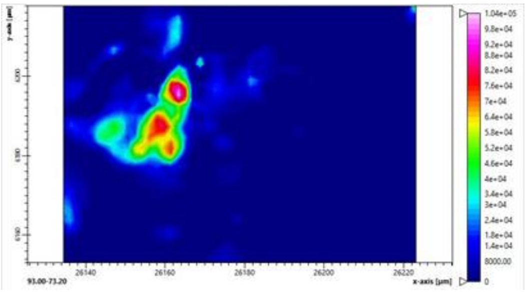	110 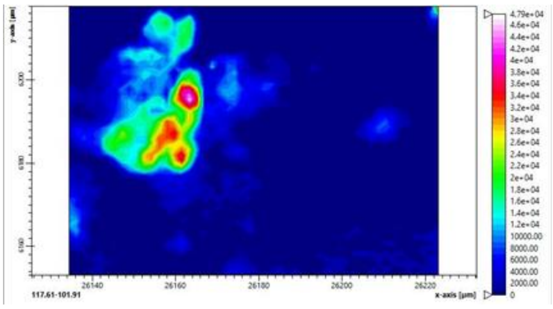
945 ThBr 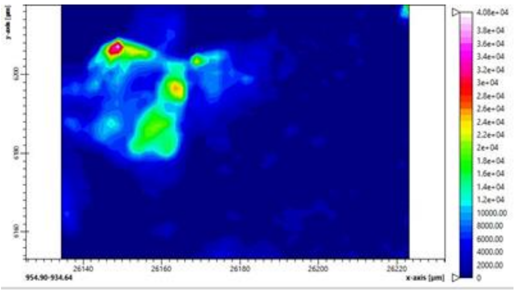	750 ThBr 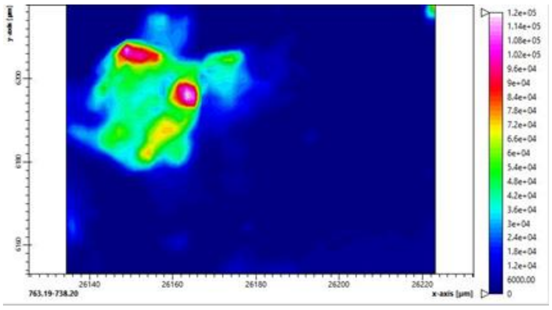
1043 str CO 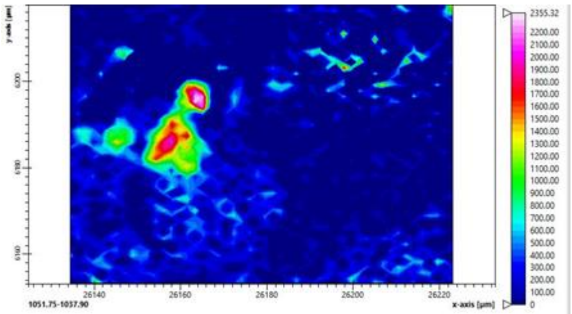	1064 str CO+strR5 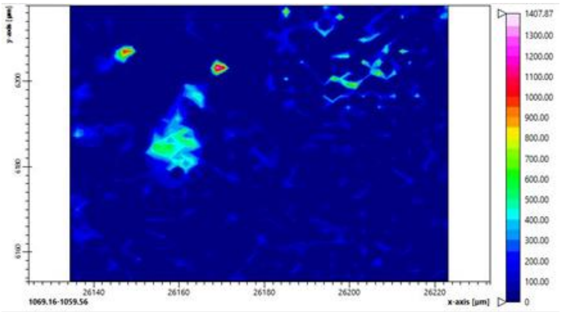
1648 ThBr 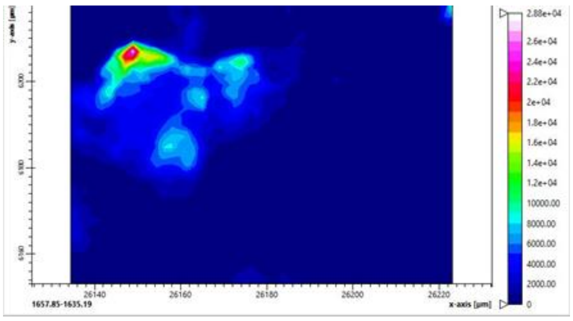	59 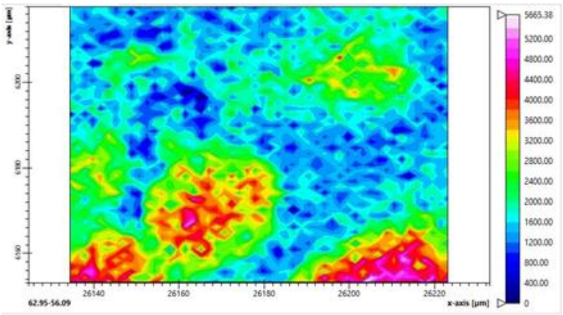

https://doi.org/10.3390/molecules31101588

PDMS 488 **Si–O–Si** symmetric stretching	PDMS 708 C-Si-C symmetric stretching
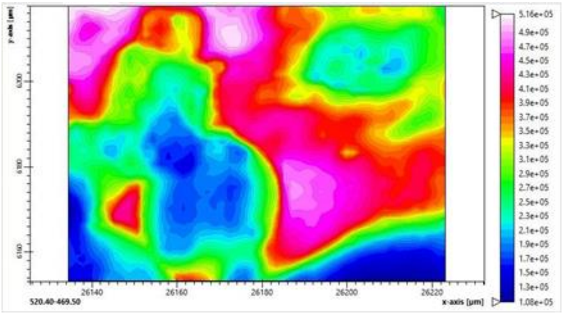	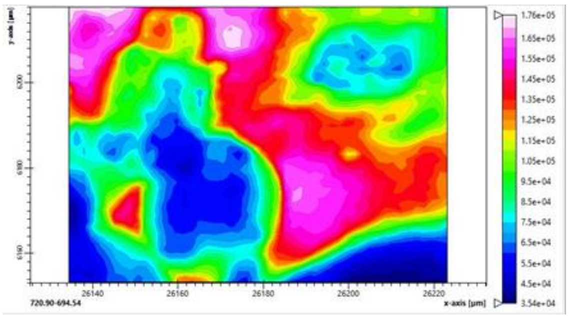

Figure S7. Raman mapping of film 26M, map 1 (air side).

In the spectra of region 26M map 1, the bands at 84 and 110 cm^{-1} in the low-frequency region (< 200 cm^{-1}), together with the C–O stretching bands at 1043 and 1064 cm^{-1}, correspond to the ThBr/NaI-P powder (Table S1). The maps of the 84 and 110 cm^{-1} bands have nearly identical patterns but differ from the maps of the other thiamine-related bands (750, 945, 1043 and 1648 cm^{-1}), which also show differences among themselves. A weak band at 59–60 cm^{-1} (seen as a shoulder on the 84 cm^{-1} band) yields a map that is very different from those of 84 cm^{-1} and of the other thiamine bands, but resembles the maps of the PDMS bands at 488 cm^{-1} (Si–O–Si symmetric stretching) and 708 cm^{-1} (C–Si–C symmetric stretching). This behaviour is consistent with the presence of Na–O–Si–O–type associates formed by NaI interacting with low-molecular hydrolysis products of TEOS (OH–Si–O–Si– species), which give an amorphous halo with a maximum at $2\theta \approx 23.1°$ in the PDMS-P XRD pattern (Section 2.3.3 of the main text) and a Raman band at 59.2 cm^{-1} (Si–O–Si scissors). Thus, in region 26M map 1 there are domains in which ThBr and NaI form crystalline associates (clusters) analogous to those observed in the ThBr/NaI-P powder.

Mapping of the third point (26M, map). (26M, map). In region 26M, map , bands at 73, 112, 1055 and 1088 cm^{-1}, characteristic of individual ThBr, and a band at 85 cm^{-1}, characteristic of the ThBr/NaI-P powder, are observed. The maps of the 73 and 112 cm^{-1} bands coincide with those of the 750 and 1648 cm^{-1} bands, whereas the maps of the C–O stretching bands at 1055 and 1088 cm^{-1} are similar to the map of the 945 cm^{-1} band. These patterns indicate the coexistence of mixed ThBr/NaI associates and individual ThBr crystallites in this region of film 26M.

**36M, map1**	
73ThBr 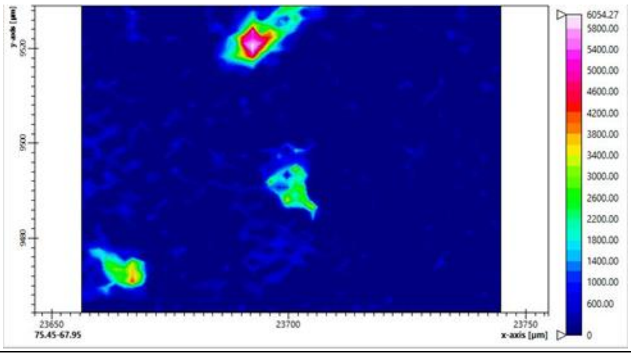	89ThBr 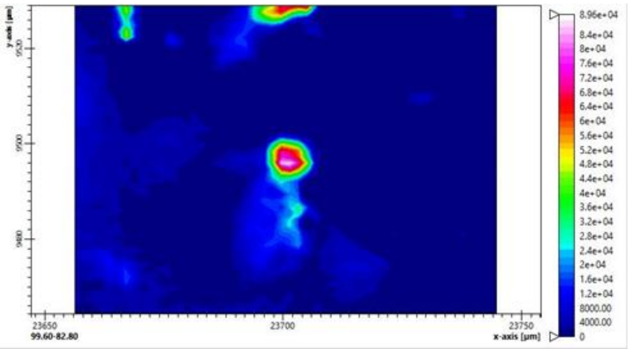
112ThBr 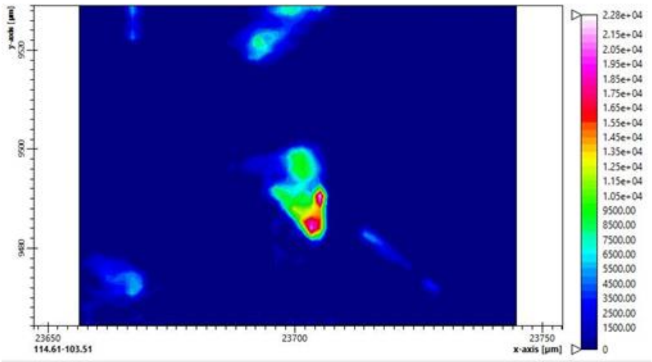	60 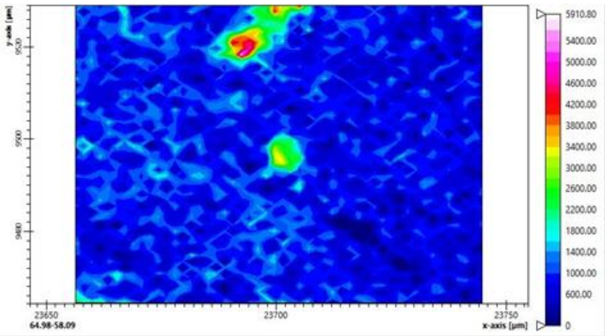
750 ThBr R6 breath 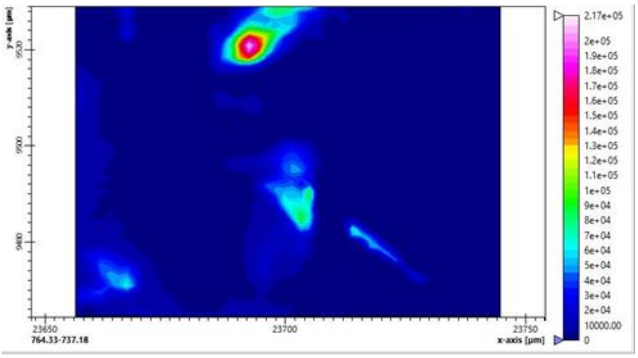	945 ThBr R5 breath 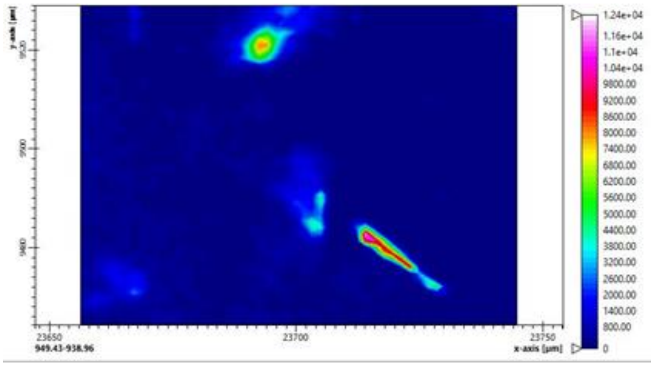
1030 ThBr 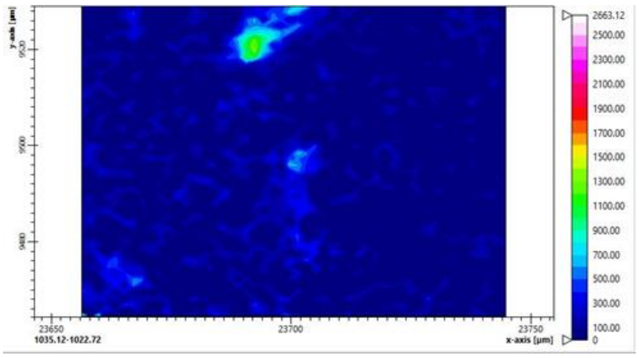	1055 ThBr CO str 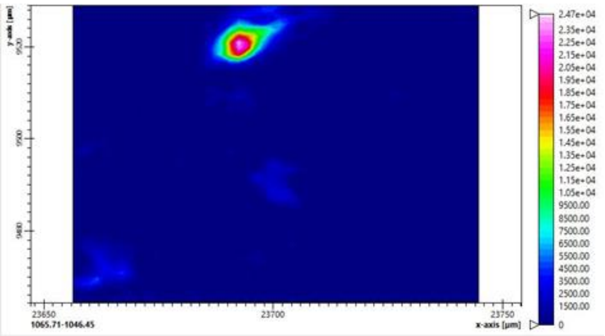
1088ThBr CO str+ R6 str	1648ThBr

https://doi.org/10.3390/molecules31101588

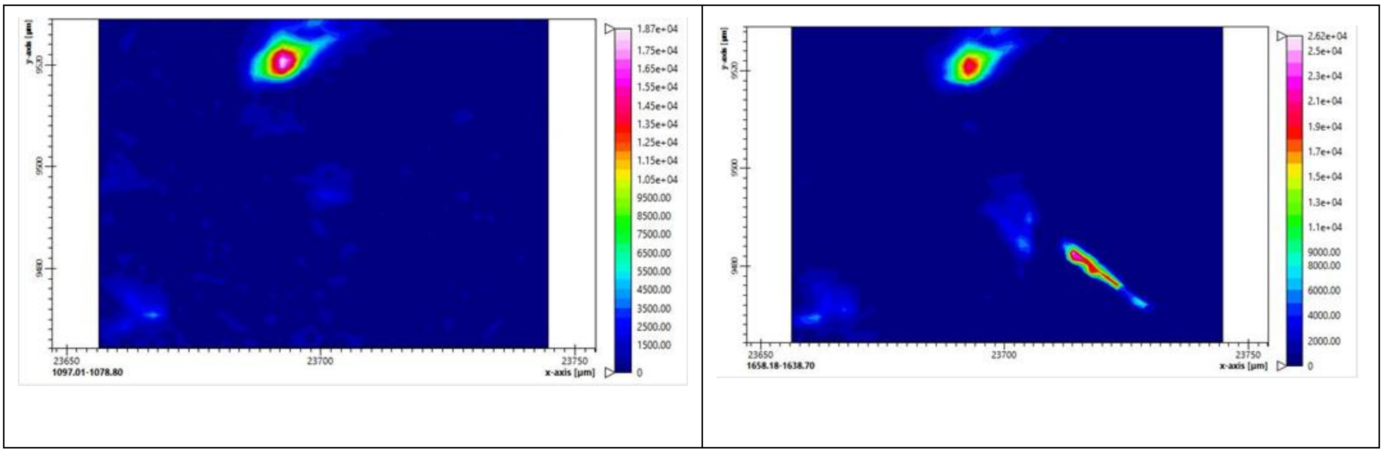

Figure S8. Raman mapping of film 36M, map 1 (air side)

In the spectra of region 36M map 1, the low-frequency bands at 60, 73, 89 and 112 cm⁻¹ correspond to individual ThBr (73.4, 89.9 and 112.3 cm⁻¹) and partially to NaI (bands at 56 and 112.3 cm⁻¹). The C–O stretching bands at 1030, 1055 and 1088 cm⁻¹ also match those of individual ThBr (1030, 1055, 1070 and 1088 cm⁻¹, Table S1). The maps of all these bands have similar patterns, indicating that in region 36M map 1 ThBr and NaI are present predominantly as individual salt crystallites rather than as mixed ThBr/NaI clusters typical of the ThBr/NaI-P powder.

In region 36M map 3, bands at 59, 73, 89, 112, 1030, 1055, 1088 and 1648 cm⁻¹ are observed, which are characteristic of individual ThBr and partly of NaI (bands at 56 and 112.3 cm⁻¹). The maps of all these bands again show similar patterns, indicating that ThBr and NaI are present mainly as individual salt crystallites rather than as mixed ThBr/NaI clusters. However, the map of the weak 59–60 cm⁻¹ band differs markedly from the maps of the other groups. Figure S9 compares the maps of the 59 and 112 cm⁻¹ bands. This difference is consistent with the contribution of Na–O–Si–O–type associates formed by NaI interacting with low-molecular hydrolysis products of TEOS (OH–Si–O–Si– species), which give an amorphous halo with a maximum at 2θ ≈ 23.1° in the PDMS-P XRD pattern and a Raman band at 59.2 cm⁻¹ (Si–O–Si scissors).

https://doi.org/10.3390/molecules31101588

59 map3	112 map3
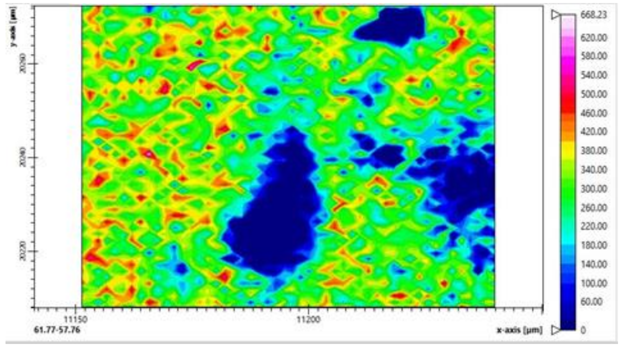	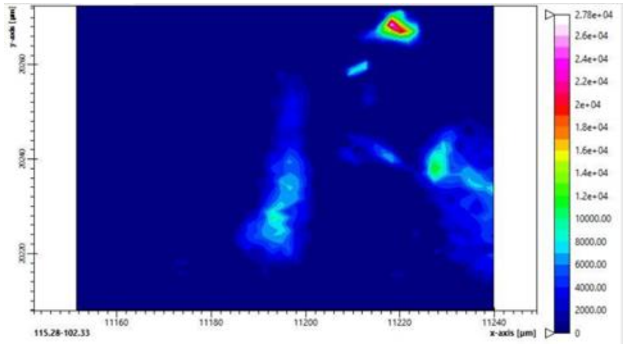

Figure S9. Raman mapping of film 36M, map 3 (bands at 59 and 112 cm⁻¹).

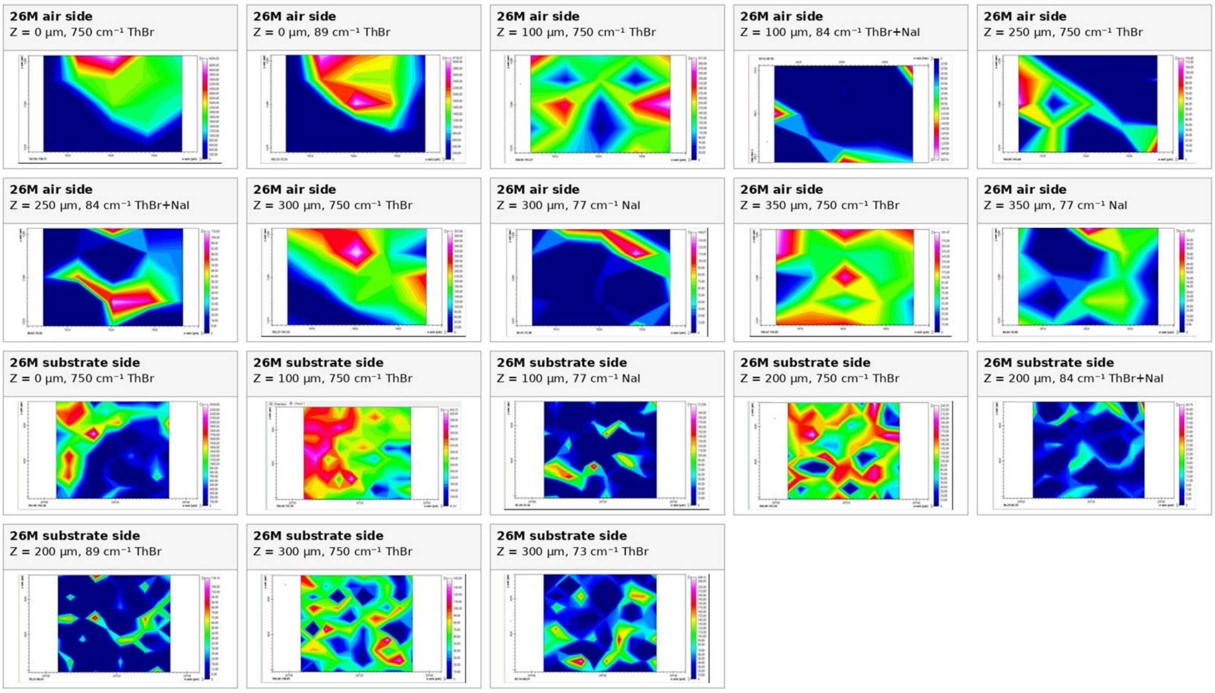

Figure S10. Complete confocal Raman depth-profiling map set of film 26M recorded from the air side and substrate side at selected depths. The maps show the spatial distribution of Raman bands assigned to ThBr-related, NaI-related and associated ThBr/NaI-type domains.

https://doi.org/10.3390/molecules31101588

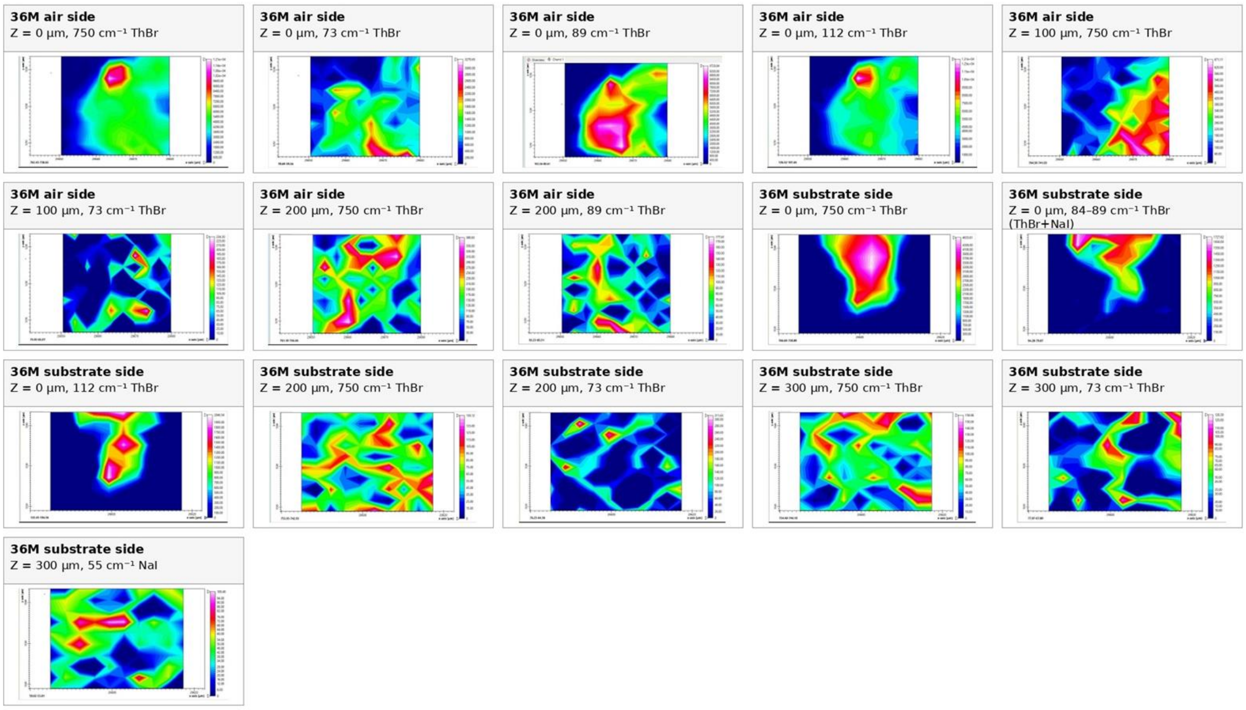

Figure S11. Complete confocal Raman depth-profiling map set of film 36M recorded from the air side and substrate side at selected depths. The maps show Raman markers associated with predominantly separated ThBr- and NaI-containing domains.

https://doi.org/10.3390/molecules31101588
